# Fumarate Reductase Activity Maintains an Energized Membrane in Anaerobic *Mycobacterium tuberculosis*

**DOI:** 10.1371/journal.ppat.1002287

**Published:** 2011-10-06

**Authors:** Shinya Watanabe, Michael Zimmermann, Michael B. Goodwin, Uwe Sauer, Clifton E. Barry, Helena I. Boshoff

**Affiliations:** 1 Tuberculosis Research Section, Laboratory of Clinical Infectious Diseases, National Institute for Allergy and Infectious Diseases, National Institutes of Health, Bethesda, Maryland, United States of America; 2 Institute of Molecular Systems Biology, ETH Zurich, Zurich, Switzerland; University of New Mexico, United States of America

## Abstract

Oxygen depletion of *Mycobacterium tuberculosis* engages the DosR regulon that coordinates an overall down-regulation of metabolism while up-regulating specific genes involved in respiration and central metabolism. We have developed a chemostat model of *M. tuberculosis* where growth rate was a function of dissolved oxygen concentration to analyze metabolic adaptation to hypoxia. A drop in dissolved oxygen concentration from 50 mmHg to 0.42 mmHg led to a 2.3 fold decrease in intracellular ATP levels with an almost 70-fold increase in the ratio of NADH/NAD^+^. This suggests that re-oxidation of this co-factor becomes limiting in the absence of a terminal electron acceptor. Upon oxygen limitation genes involved in the reverse TCA cycle were upregulated and this upregulation was associated with a significant accumulation of succinate in the extracellular milieu. We confirmed that this succinate was produced by a reversal of the TCA cycle towards the non-oxidative direction with net CO_2_ incorporation by analysis of the isotopomers of secreted succinate after feeding stable isotope (^13^C) labeled precursors. This showed that the resulting succinate retained both carbons lost during oxidative operation of the TCA cycle. Metabolomic analyses of all glycolytic and TCA cycle intermediates from ^13^C-glucose fed cells under aerobic and anaerobic conditions showed a clear reversal of isotope labeling patterns accompanying the switch from normoxic to anoxic conditions. *M. tuberculosis* encodes three potential succinate-producing enzymes including a canonical fumarate reductase which was highly upregulated under hypoxia. Knockout of *frd*, however, failed to reduce succinate accumulation and gene expression studies revealed a compensatory upregulation of two homologous enzymes. These major realignments of central metabolism are consistent with a model of oxygen-induced stasis in which an energized membrane is maintained by coupling the reductive branch of the TCA cycle to succinate secretion. This fermentative process may offer unique targets for the treatment of latent tuberculosis.

## Author Summary

Tuberculosis in its latent form infects one-third of the total human population, hiding in structures called granulomas in the lung. The dense tissue formed by the granuloma severely limits the amount of oxygen available and yet somehow the bacteria manage to survive for many years. In this study we have examined TB bacteria artificially locked at specific oxygen tensions to understand how they maintain basic metabolic functions in the absence of oxygen. As oxygen is lowered intracellular ATP levels fall and reduced cofactors such as NADH accumulate, unable to close the respiratory cycle using electron transport to donate their extra electrons to molecular oxygen. As they begin to suffocate, the bacteria flip the direction of their tricarboxylic acid (TCA) cycle enzymes from an oxidative direction to a reductive direction and begin to actively secrete succinic acid. Similar to lactate secretion in bacteria capable of growing anaerobically, this process is fermentative in nature, sufficient to enable the TB bacteria to continue basic physiologic functions like maintaining a proton gradient across the membrane, but not sufficient to allow them to grow. Understanding these processes may pinpoint vulnerabilities that could help develop interventions to treat latent infections before people develop destructive lung disease.

## Introduction

A third of the world's population is estimated to be latently infected with *Mycobacterium tuberculosis* [1]. This reservoir maintains the epidemic by ensuring the availability of future cases of reactivation disease. Any serious attempts at eradicating tuberculosis would require drastically reducing this burden of latent disease. Currently the drug of choice for prophylaxis of latent disease is isoniazid [2]. Isoniazid, which targets the cell wall by inhibiting mycolic acid biosynthesis, is thought to act on slowly or sporadically replicating *M. tuberculosis* necessitating treatment for 6–9 months to significantly reduce the risk of reactivation [3]. However, the metabolism of the mycobacteria that persist in latently infected people is poorly understood and probably not homogeneous. Recent evidence from an analysis of host transcriptional responses of latently infected individuals, compared to healthy individuals and individuals with active disease, suggests that a subset of such people are, in fact, experiencing sub-clinical disease [4]. High-resolution computed tomography (HRCT) findings in latently infected individuals likewise suggest a broad range of manifestations, ranging from enlarged lymph nodes to radiologic findings traditionally associated with active disease [5]. Tuberculosis is therefore more usefully thought of as a spectrum of disease, ranging from waning lesions in the process of being sterilized by the host immune system, through subclinical manifestations, to acute, fulminate, symptomatic infections [6]. PET/CT findings in patients with latent tuberculosis suggest that sites of infection in latently-infected individuals are hotbeds of immunologic action, with metabolic activity comparable to malignancies [7], [8], [9]. This immunologic activity is extinguished following prophylaxis with isoniazid, suggesting that bacilli in such sites are, in fact, metabolically active [10].

The hallmark of immunologic containment of *M. tuberculosis* is the formation of the granuloma. This structure develops around a core of infected macrophages surrounded by a periphery of foamy and epithelioid macrophages, monocytes and multinucleated giant cells all surrounded by lymphocytes [11]. This macroscopic structure is visible by HRCT and is present in many patients with “latent” tuberculosis (as well as in individuals with “active” tuberculosis). Activation of the infected macrophages by the lymphocytes is associated with release of reactive nitrogen intermediates which can facilitate destruction of the pathogen [12] or promote its transition to a non-replicating persistent state by inhibiting its respiration [13], [14]. The activation of lymphocytes results in further chemotaxis of immune cells, limiting the spread of disease by development of a discrete barrier wall around the infected centre. Cells within this structure can become necrotic giving a characteristic caseous central region. As granulomas mature, the periphery becomes enriched with fibroblasts that generate fibrotic material resulting in a stable structure in which the mycobacteria can persist for years [15]. The development of granulomas is associated with decreased oxygen availability that has been measured to be less than 1.6 mmHg in granulomata of *M. tuberculosis*-infected rabbits and non-human primates [16].

Hypoxia arrests the growth of *M. tuberculosis* and induces a state of non-replicating persistence through the induction of the dormancy regulon governed by DosR [17], [18]. DosR orchestrates an overall down-regulation of macromolecular metabolism. However, during this adaptation to oxygen-limiting conditions, *M. tuberculosis*: (i) switches its respiratory pathway to the less energy-efficient, higher oxygen-affinity, cytochrome bd oxidase; (ii) increases electron flow through the non-proton-pumping type II NADH dehydrogenase; and (iii) decreases expression of the F_1_F_0_-ATP synthase [19]. Drugs that target processes essential for viability of non-replicating *M. tuberculosis* are hypothesized to be particularly attractive candidates as they might have enhanced ability to sterilize lesions bearing hypoxic bacilli. Accordingly, the potent anaerobic activity of TMC207, a diarylquinoline that inhibits the F_1_F_0_-ATP synthase, is attributed to the critical role of this enzyme in maintaining the low levels of ATP characteristic of hypoxic cells [20].

The combination of severely limited oxygen availability coupled to the presence of reactive nitrogen intermediates, which inactivate respiratory cytochromes, creates a metabolic bottleneck as unrespired electrons become trapped in the form of an abundance of reduced cofactors. We have suggested that *M. tuberculosis* may use alternate electron acceptors to re-oxidize reduced cofactors and maintain an energized membrane [21]. *In vitro* models have been developed to study the metabolism of *M. tuberculosis* during hypoxic adaptation, the most popular of which is the Wayne model where the organism gradually adapts to oxygen restriction by slowing, and ultimately arresting, growth [22]. Anaerobically arrested (“persisting”) cells accumulate triacylglycerols [23], increase expression of glycine dehydrogenase and express increased nitrate reductase [22]. Although the *M. tuberculosis* nitrate reductase genetically contains all the elements of a proton pumping respiratory enzyme, nitrate appears not to support growth of this organism and the function of this enzyme may be limited to a role in nitrogen assimilation [24]. Not surprisingly, anaerobic *M. tuberculosis* shows a dramatic increase in its NADH/NAD^+^ ratio [25]. Transcriptional profiling of microaerophilic and anaerobic *M. tuberculosis* cells showed increased expression of malic enzyme and fumarate reductase with concomitant downregulation of other TCA cycle genes such as citrate synthase and isocitrate dehydrogenase [25], [26], [27]. We have previously hypothesized that *M. tuberculosis* may re-oxidize reducing equivalents while maintaining an energized membrane by operating the second half of the TCA cycle in a reductive direction [21].

In the present work, we have developed a chemostat model for *M. tuberculosis* to allow growth at well defined dissolved oxygen concentrations in order to analyze the metabolism of *M. tuberculosis* in response to precise levels of this external electron acceptor. We show that succinate accumulates in supernatants of hypoxic cultures and confirm that this arises through the second half of the TCA cycle operating in the reductive direction by isotopomer analysis and metabolomics after stable isotope feeding experiments.

## Results

### *M. tuberculosis* growth rate adapts to dissolved oxygen concentration

**Figure 1:**
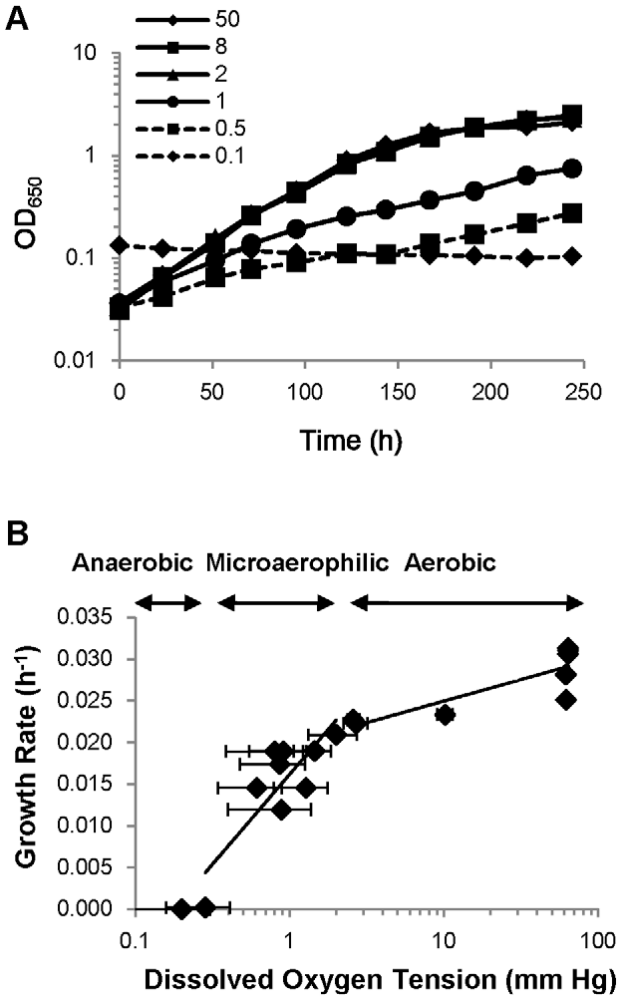
Oxygen concentration directly affects the growth of *M. tuberculosis*. (A) Growth curve of H_37_Rv in fermentor culture maintained at various dissolved oxygen tensions. H_37_Rv cultures were grown in six fermentors and growth was followed by measuring OD_650_. 10 ml (50, 8, 2, 1 and 0.5 mmHg culture) or 50 ml (0.1 mmHg culture) of H_37_Rv preculture (OD_650_ = 0.26) was inoculated in 850 ml of Dubos medium in fermentors. They were preincubated for 3 days to reach each set point of dissolved oxygen concentrations after which the measurements were recorded as shown. (B) Growth rate of H_37_Rv under defined oxygen tensions. Growth rate was calculated from the growth curve of H_37_Rv culture in Dubos medium in a fermentor.

*M. tuberculosis* replicates under aerobic conditions but can survive extended periods of time anaerobically in the absence of replication [22]. We have previously reported that the growth rate of the organism can be modulated by passing a defined flow of decreasing oxygen concentrations over the headspace of a sealed flask [28]. Unfortunately, the growth rate in this model is proportional to the flow and the actual dissolved oxygen concentration is undefined. In the Wayne model of *M. tuberculosis* adaptation to hypoxia [22], oxygen depletion is induced by growth and the rate of decrease, while reproducible, does not allow continuous interrogation of the metabolism of *M. tuberculosis* at a specific oxygen tension. We therefore developed a fermentor model of *M. tuberculosis* growth where controlled oxygen levels were achieved using a 3-day gradient to a final desired oxygen concentration ranging from normoxic to hypoxemic. Figure 1A shows the growth curves of *M. tuberculosis* H_37_Rv in Dubos medium under 6 different dissolved oxygen tensions and Figure 1B shows the extrapolated growth rate of H_37_Rv under these dissolved oxygen tensions. We could identify three different stages based on the growth rate as a function of oxygen tension. In the first aerobic stage (from 50 to 2 mmHg dissolved oxygen tension), the growth rate decreased only slightly from 0.028 h^−1^ to 0.021 h^−1^. In the second microaerophilic stage (from 1 mm to 0.28 mmHg dissolved oxygen tension), there was a sharp inflection in the doubling time ultimately decreasing to approximately 0.0002 h^−1^. In the largely anoxic stage below 0.28 mmHg, *M. tuberculosis* growth was completely arrested. This corresponds to the end state of non-replicating persistence in Wayne model cells. Interestingly, we noted that anaerobic growth of *M. tuberculosis* was associated with acidification of the medium requiring continual automatic adjustment of the pH of the medium in the fermentor with 1 N NaOH solution whereas addition of 1 N HCl was needed for growth above 2 mmHg to maintain constant pH.

### NADH/NAD^+^ ratio and ATP synthesis is a function of dissolved oxygen concentration

**Figure 2:**
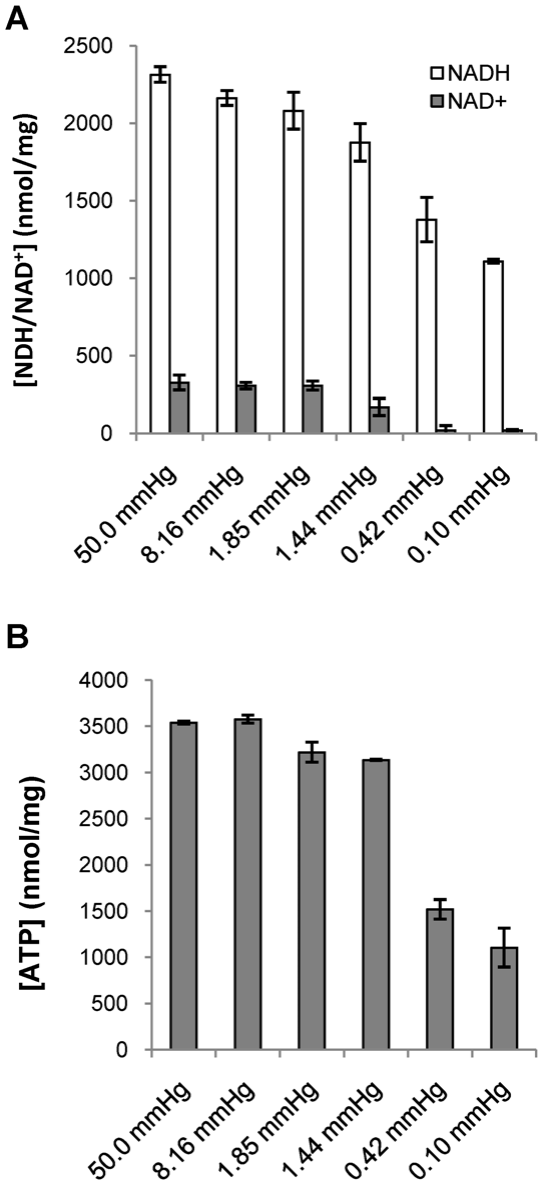
NADH/NAD^+^ and ATP concentration is a function of dissolved oxygen concentration. (A) NADH and NAD^+^ and (B) ATP concentrations under various dissolved oxygen tensions. NADH/NAD^+^ and ATP concentrations were measured in chemostat cultures with a constant growth rate of 0.0077 h^−1^ under 50.0, 8.19, 1.85, 1.44 and 0.42 mmHg of dissolved oxygen tensions. They were also measured in batch culture under 0.10 mmHg on Day 10.

To explore oxygen tension-dependent changes in metabolism, NADH/NAD^+^ concentrations were measured in cell pellets isolated from fermentor cultures grown under these defined dissolved oxygen tensions. In chemostat culture the levels of NADH/NAD^+^ were determined at a constant growth rate set by adjusting feeding rates of both oxygen and growth medium. Once the cultures reached constant biomass, optical density and bacillary numbers at a growth rate of 0.0077 h^−1^, the NADH/NAD^+^ levels were determined. At lower oxygen tensions there was no growth. Thus for 0.1 mmHg cultures, NADH/NAD^+^ levels were determined 10 days after reaching the set oxygen concentration. These analyses revealed that NADH/NAD^+^ ratios and the total combined concentration of this cofactor were unperturbed by changes in oxygen tensions from 50 – 1.85 mmHg whereas the reduced to oxidized ratio increased from 7 to 11 at 1.44 mmHg concomitant with an absolute decrease in cofactor concentration (Figure 2A). At oxygen tensions of 0.42 mmHg and below, the reduced to oxidized ratio increased dramatically (Table 1). Consistent with previous reports, as oxygen tension was decreased, intracellular ATP levels declined significantly (Figure 2B) with intracellular levels dramatically reduced at oxygen tensions of 0.42 mmHg and lower.

**Table 1:** NADH/NAD^+^ ratio under various dissolved oxygen tensions.

DOT (mmHg)	NADH/NAD^+^	SD
50.0 mmHg	7.12	1.15
8.16 mmHg	7.09	0.59
1.85 mmHg	6.81	0.32
1.44 mmHg	11.1	3.96
0.42 mmHg	69.4	8.12
0.10 mmHg	56.1	9.10

### Transcriptional analysis of *M. tuberculosis* during growth at reduced oxygen tensions

Inspection of previously published microarray data on Wayne-model adapted cells implicated the upregulation of several enzymes of the reductive branch of the TCA cycle. In particular, fumarate reductase, phosphoenolpyruvate carboxykinase and malic enzymes were found to be strongly upregulated in microaerophilic and anaerobic cultures [25], [26], [27], [29]. Notably, the latter two directly link glycolysis to the TCA cycle. In contrast, components of the pyruvate dehydrogenase complex (*aceE*, *pdhA*), citrate synthase and a putative α-ketoglutarate decarboxylase (*kgd* [30], also annotated as a 2-hydroxy-3-oxoadipate synthase [31]) of the oxidative TCA branch were downregulated [25], [26], [27], [29]. Isocitrate dehydrogenase, which controls the levels of isocitrate available to the glyoxylate shunt as well as the TCA cycle, and isocitrate lyase have also been reported to be upregulated under hypoxia [25], [26], [27], [29]. Taken together, these results suggested that the enzymes of the reductive half of the TCA cycle may play an important role as cells adapt to hypoxia leading us to hypothesize that reduced cofactors could be re-oxidized by fermentation.

To confirm these observations, we analyzed the expression of selected TCA cycle genes potentially involved in succinate formation by quantitative RT-PCR from chemostat cultures adapted to defined oxygen concentrations (Figure S1 and Figure S2). As positive controls, the dormancy regulon genes *tgs* and *hspX* were also monitored and were, as expected, highly upregulated even at oxygen concentrations that did not dramatically affect the NADH/NAD^+^ ratios or ATP levels (Figure S1 and Figure S2). Notably, enzymes involved in the oxidative direction of the TCA cycle were downregulated significantly, including citrate synthase (*citA*), aconitase (*acn*), and α-ketoglutarate dehydrogenase (*kgd*). *M. tuberculosis* encodes three fumarate oxidoreductase/succinate dehydrogenase homologs that could directly give rise to succinate formation including *frdABCD* (Rv1552–Rv1555), *sdhCDAB* (Rv3316–Rv3319) and Rv0247c-Rv0249c. We observed that *frdA*, the annotated fumarate reductase, was upregulated 212-fold in hypoxic non-replicating cultures maintained at 0.1 mmHg whereas its functional homologs, *sdhA* and Rv0248c, were both slightly downregulated (Figure S2).

**Figure 3:**
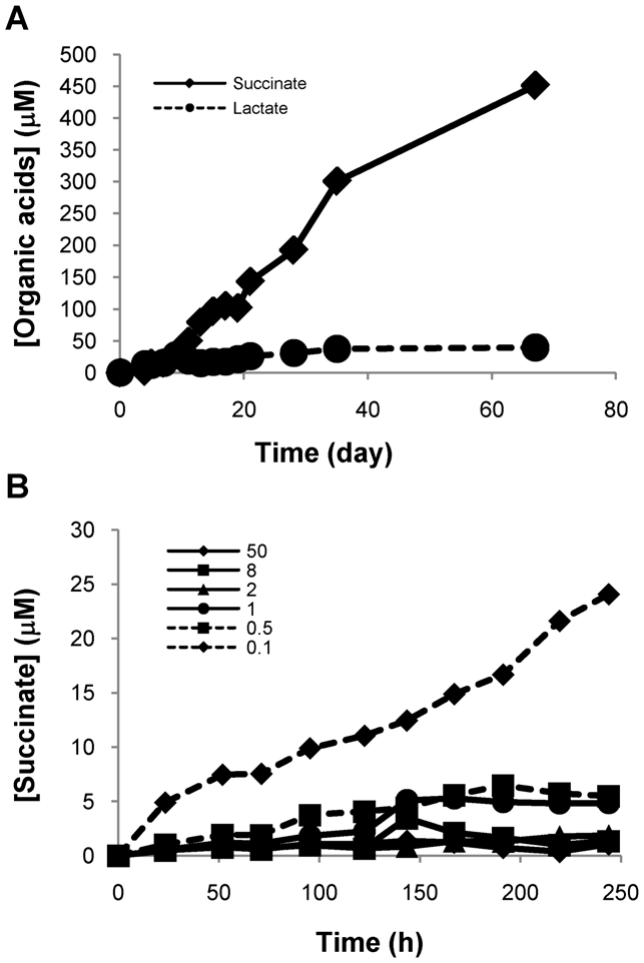
Succinate accumulates in supernatants of H_37_Rv culture under anaerobic condition. (A) Succinate and lactate accumulations in NRP culture. H_37_Rv was grown in Wayne tubes with septa and the supernatant was collected via syringe. Succinate and lactate concentrations were determined by LC/MS. (B) Succinate accumulation in supernatants under various dissolved oxygen tensions. Succinate concentrations were measured by LC/MS in supernatants of the H_37_Rv cultures shown in Figure 1A.

Operation of the TCA cycle in this direction requires assimilation of CO_2_ into pyruvate either through pyruvate carboxylase (*pca*) to form oxaloacetate or through malic enzyme (*mez*) to form malate. We therefore also examined the expression of these enzymes in the presence and absence of CO_2_. We found that malic enzyme (*mez*) was downregulated in the absence of CO_2_ but slightly upregulated in cultures grown in its presence. Phosphoenolpyruvate carboxykinase (*pckA*) was also slightly upregulated during growth at reduced oxygen concentrations in a CO_2_-dependent fashion whereas another potentially CO_2_-fixing enzyme (pyruvate carboxylase) was downregulated (Figure S2 and Figure S3). Although *pckA* was upregulated in cultures adapted to hypoxia in the Wayne model, *mez* was not, perhaps reflecting differences in CO_2_ levels achieved in Wayne model cultures or repression by other signals in these cultures (Figure S2). Consistent with our finding that lactate also slightly accumulated during anaerobic non-replicating persistence (Figure 3A; *vide infra*), lactate dehydrogenase, was upregulated by reduced oxygen concentrations (Figure S1 and Figure S2).

### Anaerobic adaptation of *M. tuberculosis* is coupled to succinate secretion

We have previously developed methodology to quantitate the organic acids of the TCA cycle from the supernatant of *M. tuberculosis* cultures using ion exclusion chromatography [32]. Because of our observation of acidification of the media, we measured succinate, pyruvate, malate, lactate and fumarate concentrations in aerobically growing and anaerobically adapted cells first using the Wayne model (Figure 3A and Table S1). In the Wayne model, cells progress through two stages of non-replicating persistence (NRP), NRP-1 and NRP-2, with the transition into NRP-1 occurring once the exponentially growing cells have consumed oxygen to a level of 1 mmHg and NRP-2 being reached when oxygen levels are reduced to less than 0.09 mmHg. We observed a significant accumulation of succinate during adaptation to microaerophilic and anaerobic conditions that was not observed in aerobically growing cultures at similar or higher cell densities (Figure 3A, Table S1). Succinate levels in the supernatant of NRP-2 cultures accumulated to nearly 500 µM while lactate levels increased only slightly during anaerobic persistence (Figure 3A and Table S1). We then employed our fermentor system to better define the transition from respiratory to fermentative metabolism by measuring succinate concentrations in the supernatant under defined oxygen tensions. Succinate accumulation coincided primarily with the onset of anaerobic conditions and the cessation of growth. Small amounts of succinate accumulated in the supernatant of H_37_Rv culture maintained at 1.0 and 0.5 mmHg O_2_ but was maximal at 0.1 mmHg (Figure 3B). To rule out that the observed succinate was accumulating in response to nitrogen assimilation from asparagine we repeated the analysis with media in which asparagine was replaced with aspartate and observed the same robust accumulation of succinate.

### Stable isotope labeling studies support the reductive TCA cycle

**Figure 4:**
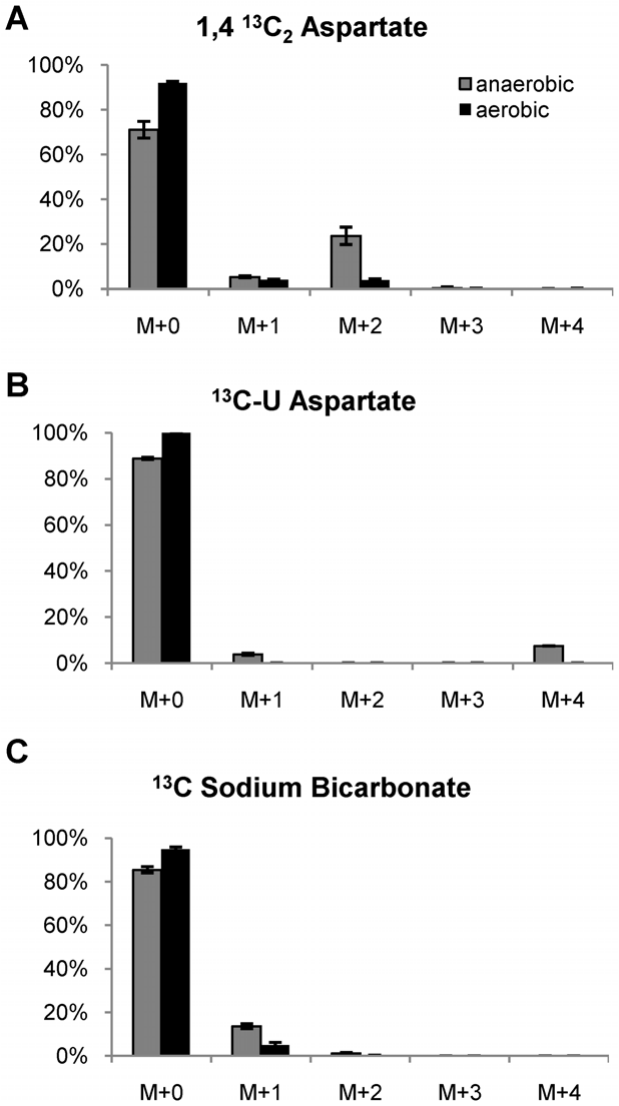
Mass isotopomer ratios of secreted succinate following labeling with ^13^C isotopic substrates. H_37_Rv was grown in a Wayne model tube for 5 days to adapt hypoxic conditions and then exposed to (A) [1,4-^13^C_2_]- aspartate, (B) U-^13^C aspartate and (C) ^13^C sodium bicarbonate in an anaerobic chamber for 24 h. The results of anaerobic conditions were compared with those from aerobically growing cultures.

To confirm the role of the reductive branch of the TCA cycle under hypoxia, we monitored the fate of stable isotope-labeled substrates in these cultures. In the oxidative branch of the TCA cycle, two CO_2_ molecules are lost corresponding to the C1 and C4 positions of oxaloacetate. If the observed succinate accumulation was due to the TCA cycle functioning in the oxidative direction, substrates such as [1,4-^13^C]aspartate, which is transaminated to [1,4-^13^C]oxaloacetate, would be incorporated into succinate by loss of the corresponding 1-^13^C and 4-^13^C carbons and the resulting succinate would have a mass corresponding to the ^12^C_4_ isotopomer. On the other hand, if the succinate was formed by the reductive branch, the resulting molecule would retain both of these carbons and be enriched for an M+2 isotopomer. Figure 4A shows the mass isotopomer ratios of succinate in the culture supernatant following labeling with [1,4-^13^C]aspartate. Aerobic growth in Dubos medium in the presence of an excess of [1, 4-^13^C]-L-aspartate, resulted in only small amounts (3.8%) of the M+2 isotopomer in secreted succinate (Figure 4A). However, similar labeling experiments under anaerobic conditions resulted in significant accumulation of doubly ^13^C-labeled succinate (23.5% of total succinate) (Figure 4A). These results were further confirmed by growth on [U-^13^C]aspartate, which resulted in secretion of quadruple labeled succinate (M+4) in anaerobic supernatants with no measurable succinate M+4 isotopomer detected in aerobic supernatants (Figure 4B).

**Figure 5:**
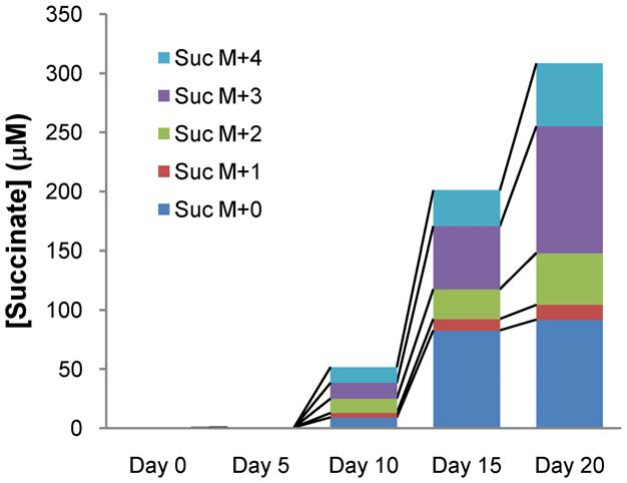
Isotopic succinate accumulation in supernatant of NRP culture in 0.75% U-^13^C Glucose Dubos medium. H_37_Rv was grown in Dubos medium where glucose was replaced with U- ^13^C glucose using the Wayne model of hypoxic adaptation. The supernatant was collected every 5 days and secreted succinate was analyzed for ^13^C incorporation. These cells reach NRP-1 by day 10 and NRP-2 by day 15, coincident with a significant increase in the M+3 fraction of secreted succinate arising from reversal of the TCA cycle.

The observed upregulation of isocitrate lyase under hypoxia (Figure S1 and Figure S2) suggested that succinate accumulation could also have been driven by other metabolic pathways. Metabolism of a substrate such as [U-^13^C]glucose to acetyl-CoA followed by conversion to succinate by the glyxolyate shunt or through operation of the oxidative branch of the TCA cycle would result in accumulation of the M+2 isotopomer of succinate. Alternatively, the M+3 isotopomer would accumulate if glucose fed into the reductive branch of the TCA cycle through malic enzyme and/or phosphoenolpyruvate carboxykinase. To elucidate the potential usage of the glyoxylate shunt or the reductive TCA, we analyzed sequential supernatants of *M. tuberculosis* during hypoxic adaptation in Wayne model cultures wherein the glucose was replaced with [U-^13^C]glucose every five days. These results showed that dramatic succinate accumulation occurred by day 10 when the cells had reached NRP-1. From that time forward we observed an increase in the M+0 fraction, likely driven by the asparagine in the Dubos medium replenishing oxaloacetate in the TCA cycle. Coincident with this change, the M+3 fraction expanded rapidly, although some M+2 succinate isotopomer continued to be formed (Figure 5). The relative difference in the M+3 fraction was especially marked at after day 15 when the cells had reached NRP-2 and this isotopomer accounted for 34.7% of total secreted succinate by day 20. This indicates a switch in metabolism in the reductive branch of TCA cycle coincident with the onset of anaerobic conditions. The CO_2_-dependent upregulation of malic enzyme and phosphoenolpyruvate carboxykinase suggested that intermediates of the reductive TCA branch are replenished by carbon fixation. To test this feeding experiments with H^13^CO_3_ were performed and showed a significant increase in the succinate M+1 isotopomer fraction in anaerobic supernatants (Figure 4C).

**Figure 6:**
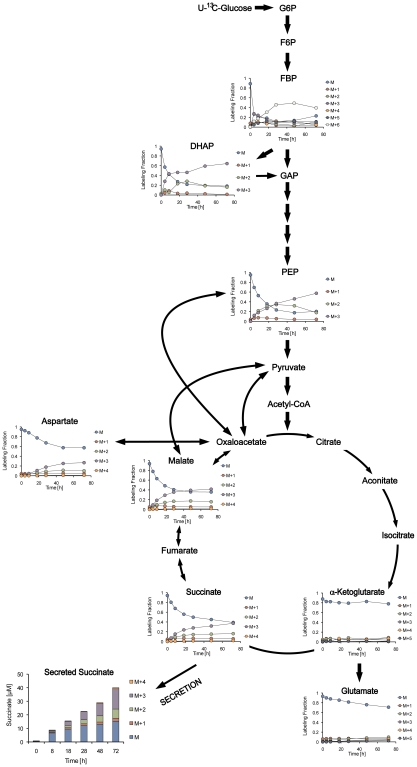
Dynamic isotopic intracellular metabolite measurements in anaerobically persisting cells during metabolism of U-^13^C glucose. The schematic shows isotopomer distribution of intracellular glycolytic and TCA cycle intermediates during metabolism of U-^13^C glucose under anaerobic conditions following replacement of the glucose with U-^13^C glucose before metabolomics analyses over time. Under anaerobic conditions the isotopomer distribution of the intracellular metabolites support flux towards the reverse TCA cycle and mirror the isotopomer distribution of the secreted succinate.

**Figure 7:**
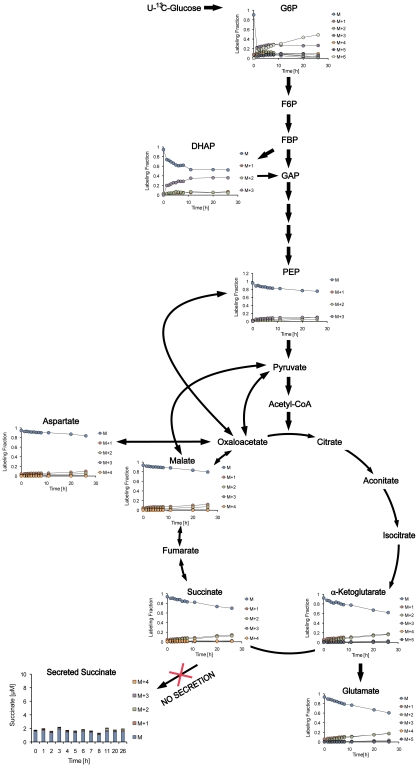
Dynamic isotopic intracellular metabolite measurements in aerobic growing cells during metabolism of U-^13^C glucose. Schematic illustration of glycolysis and the TCA cycle and graphs of isotopomer distribution during metabolism of U-^13^C glucose. H_37_Rv was grown under aerobic conditions in Dubos medium where glucose was replaced with U-^13^C glucose followed by metabolomics analyses over time. Under aerobic conditions the isotopomer distribution supports forward flux through the oxidative TCA.

To confirm the operation of the reductive branch of the TCA cycle in anaerobically persisting cells, we performed dynamic labeling experiments of anaerobically adapted cells where the glucose was replaced under anaerobic conditions with [U-^13^C]glucose. We then measured all metabolites involved in glycolysis and the TCA cycle and compared these to the same metabolites formed in aerobically growing cells. These results showed that the reverse branch of the TCA cycle became enriched with the M+3 isotopomer of the corresponding metabolites under anaerobic conditions, supporting the hypothesis that metabolites from glycolysis fed directly into the C4 branch of the TCA cycle (Figure 6 and Figure S4). In contrast, under aerobic conditions, no M+3 isotopomers of metabolites of the TCA cycle were detected, only the M+1 and M+2 isotopomers that would be expected during oxidative decarboxylation of pyruvate (Figure 7).

### Fumarate reductase activity increases during anaerobic persistence

**Figure 8:**
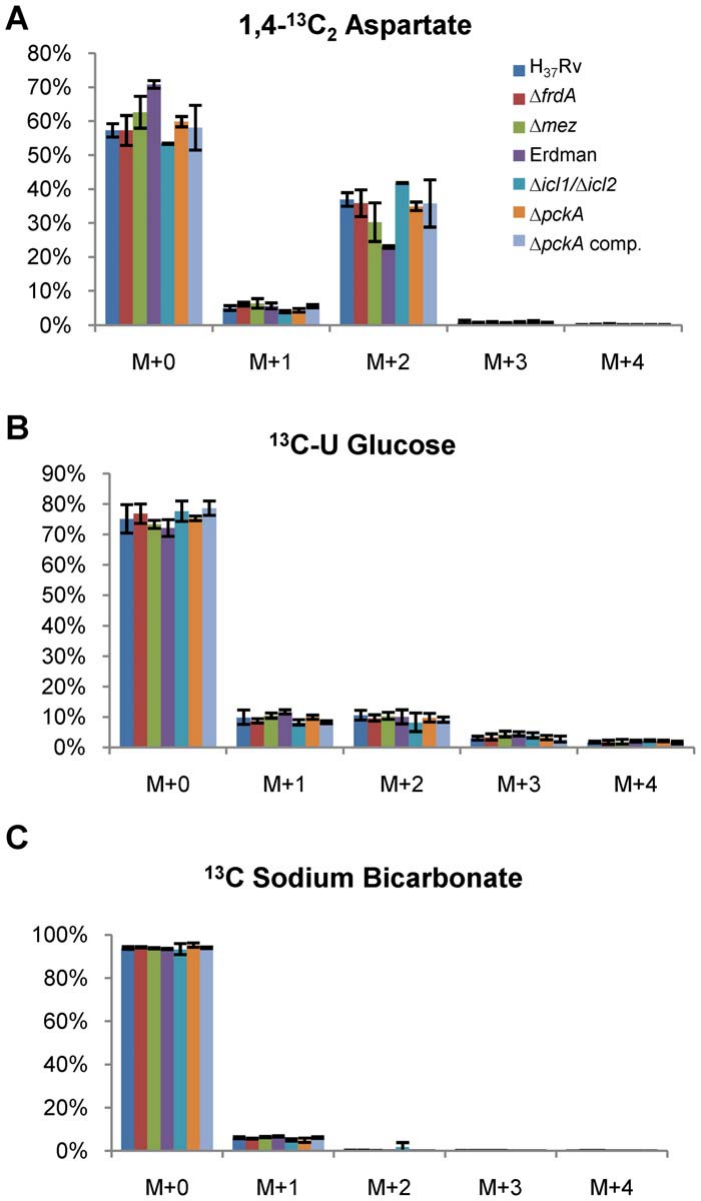
The mass isotopomer ratios of succinate in supernatant of knock out mutant strains labeling with ^13^C isotopic substrates. Cultures of H_37_Rv Δ*frdA*, H_37_Rv Δ*mez*, Erdman Δ*icl1*/Δ*icl2* and Erdman Δ*pckA* as well as their parent strains and complement strain of Erdman *pckA* were exposed to (A) 1,4-^13^C_2_ aspartate, (B) U-^13^C glucose and (C) ^13^C sodium bicarbonate in an anaerobic chamber for 24 h. Ratios of succinate isotopomers were measured by LC/MS.

The role of fumarate reductase, malic enzyme and phosphoenolpyruvate carboxykinase in the reverse TCA cycle were explored by creating allelic exchange mutants of *frdA* and *mez* and by obtaining a mutant of *pckA* [33]. Surprisingly, none of the mutants showed any significant survival defect upon entry into anaerobiosis in the Wayne model nor upon non-replicating survival in the fermentor model out to 10 days under 0.1 mmHg dissolved oxygen tension (data not shown). In addition, the contribution of isocitrate lyase to any possible succinate accumulation was explored with the *icl1*/*icl2* double knockout mutant compared to its parental strain (Erdman) since previous studies have indicated the importance of the glyoxylate shunt during slow growth and adaptation to non-replicating persistence [34], [35]. To explore the role of these enzymes in succinate accumulation, we repeated the stable isotope labeling with [1,4-^13^C]aspartate, [U-^13^C]glucose and H^13^CO_3_ with each of these mutant strains. There was no significant difference between the mutants and their corresponding wild type parental strains (Figure 8) in the isotopomer distribution of succinate formed during anaerobiosis.

The absence of a phenotype in the *frdA* mutant suggested the possibility that the succinate/fumarate dehydrogenases encoded by *sdhCDAB* (Rv3316–Rv3319) and Rv0247c-Rv0249c could have compensated for the loss of this activity. These enzymes can all reversibly interconvert fumarate and succinate although they have been reported to have different equilibrium constants with respect to their bidirectionality [36], [37] Although these enzymes were not upregulated under hypoxia in the wild type strain (Figure S2), their relative expression levels were higher in the mutant than in the wild type strain (Figure S2). Likewise, in the *mez* and *pckA* knockout mutants, compensatory increased expression of the alternative enzymes (pyruvate carboxylase, malic enzyme or phosphoenolpyruvate carboxykinase) linking the TCA cycle to glycolysis was observed (Figure S2). To directly assess the alternative succinate/fumarate dehydrogenases in mutants lacking *frdA*, we determined succinate dehydrogenase activities in membrane fractions from aerobic as well as from 3-week anaerobically adapted Wayne model cells. These results showed that the specific enzyme activities between the parental and *frdA* knockout strains were very similar under aerobic and anaerobic conditions (Figure S5). Surprisingly, the specific enzyme activities in membrane fractions from aerobic as opposed to anaerobic cells were not dramatically different despite the more than 100-fold transcriptional upregulation observed for *frdA* under anaerobic conditions.

### Fumarate reductase genes are not essential for in vivo pathogenesis in the mouse

Transcriptional profiling of *M. tuberculosis* during growth in murine bone marrow macrophages has indicated that the *frdABCD* gene cluster is upregulated in activated but not resting host cells in a nitric oxide independent manner whereas the succinate dehydrogenase gene clusters encoded by *sdhCDAB* and Rv0247c-Rv0249c genes were not differentially regulated [14]. A study of *M. tuberculosis* gene expression signatures obtained from a variety of different lung lesions from tuberculosis patients has also shown that the genes of the fumarate reductase/succinate dehydrogenase gene clusters were upregulated in lung sections distant from the granuloma in comparison to expression within the granuloma. These studies failed to conclusively show differences however, due to absence of co-regulation of genes within the clusters (and even inverse expression of other members of this operon) making definite conclusions about the functional consequences of these signatures impossible [38] Transcriptional analyses of *M. tuberculosis* derived from patient sputum indicated that genes of the Rv0247c-Rv0249c gene cluster were downregulated with no evidence of regulation of the other succinate/fumarate dehydrogenase genes in this environment [39]. These results suggest that the fumarate reductase/succinate dehydrogenase genes are differentially regulated in the variety of microenvironments encountered in the host *in vivo*. An *in vivo* analysis of a genome-wide transposon mutant library indicated that the Rv0247c-Rv0249c gene cluster may play a role during growth in infected mice as seen by the attenuation of the Rv0249c transposon mutant during the course of infection whereas the *frdABCD* and *sdhCDAB* genes were not essential up to 8 weeks in infected mice.

To test the essentiality of the fumarate reductase in the metabolism of *M. tuberculosis* during murine infection, we aerosol infected C57Bl/6 mice with the *frdA* knockout mutant as well as the parental H37Rv. Groups of five mice were harvested at five time points following infection and the bacillary burden in their lungs and spleens enumerated. These results showed that there was no difference in the rate of replication, nor in persisitence after replication had been contained in the organs of mice infected with the *frdA* mutant as compared to the wild type strain (Figure S6) confirming that this gene is not essential for murine pathogenesis.

## Discussion

*M. tuberculosis* is able to persist in a non-replicating state induced by oxygen restriction or low concentrations of nitric oxide [13]. It is thought that respiratory arrest may be a significant factor during disease since slow or non-replicating organisms display phenotypic drug tolerance and may persist in infected humans without showing signs of fulminant disease [6], [21]. We have demonstrated that, not surprisingly, the growth rate of *M. tuberculosis* is a direct function of dissolved oxygen tension. In the Wayne model where the actual oxygen concentrations are not known, two stages of non-replicating persistence are observed depending on the oxygen concentration [22]. At 1% dissolved oxygen concentration (corresponding to 1.5 mmHg) growth rates are slowed but the absorbance of the culture still increases. At 0.06% dissolved oxygen concentration (0.09 mmHg) no further increase in absorbance is observed. Similarly we have found that the growth rate of *M. tuberculosis* in a chemostat under oxystatic conditions can be modeled as 2 distinct phases with microaerophilic growth occurring at dissolved oxygen tensions less than 2 mmHg at between 0.015–0.025 generations per hour. Anaerobic persistence, however, without actual growth, appears to occur at oxygen tensions of less than 0.2 mmHg. Thus the transition from aerobic to microaerophilic growth (or between NRP-1 and NRP-2 in the Wayne model) appears to correspond to a threshold of between 1 and 2 mmHg of oxygen, notably close to the 1.6 mmHg oxygen tension we measured in caseous granulomas within the lungs of infected rabbits [16]. This suggests that this growth transition may have direct physiological relevance and perhaps has evolved in response to a need of the organism to adapt to the microaerophilic conditions that prevail in the tuberculous granuloma.

In *M. tuberculosis* the reduced availability of a terminal electron acceptor is associated with an increase in the NADH/NAD^+^ ratio indicating a reduced capacity for reoxidation of this cofactor. The reduced capacity for NADH reoxidation paralleled the changes in growth rate and became most apparent at oxygen concentrations below 1.85 mmHg. Concomitant with this decreased metabolism intracellular ATP levels dropped. It has previously been demonstrated that ATP levels are sustained at a critically low level in anaerobic cultures [20] by the F_1_F_0_-ATP synthase. ATP synthesis through this enzyme, however, requires maintenance of a proton gradient across the membrane, and while reduced cofactors could perhaps be reoxidized during the triacylglycerol synthesis that accompanies adaptation to critically low levels of oxygen [23], this oxidation does not contribute to the generation of a proton motive force. In addition, the upregulation of the DosR regulon at oxygen tensions that supported rapid growth, suggests that TAG synthesis controlled by the DosR-regulated triacylglycerol synthases does not play a critical role in the metabolism that distinguishes actively replicating aerobic from hypoxically restricted persistent cells. Therefore, when we observed an unusual acidification of cultures maintained anaerobically we began to search for secreted acids and identified a striking accumulation of secreted succinic acid in anaerobic cultures in both fermentor and Wayne models of non-replicating persistence. Succinate secretion is common in obligately anaerobic bacteria, and widespread amongst filamentous fungi but has not been previously reported for supposedly obligate aerobes such as *M. tuberculosis* [40]. The efflux of succinate is electrogenic and bioenergetic studies in *E. coli* have shown that H+/succinate symport is very efficient [41].

Analysis of gene expression levels of *M. tuberculosis* during survival under oxygen tensions that do not support replication previously revealed upregulation of phosphoenolpyruvate carboxykinase and the malic enzyme which catalyzes the oxidative decarboxylation of malate coupled with the reduction of NAD(P)^+^ producing pyruvate and CO_2_ [42]. Malic enzyme has been associated with increased activity through the reductive branch of the TCA cycle [43]. This enzyme is also highly upregulated under anaerobic conditions in *Salmonella* spp [44]. However, malic enzyme may also function in the reverse direction in organisms such as *E. coli* and *Chlamydomonas reinhardtii* to increase metabolites in the C4 branch of the TCA cycle producing succinate by fermentation during anaerobic growth in the absence of hydrogenase activity of this organism [45], [46], [47]. Alternatively, supplementing cellular pools of terminal electron acceptors in the C4 branch of the TCA cycle can also be done by reversing the direction of phosphoenolpyruvate carboxykinase reaction [48]. At this stage we do not have evidence that either of these enzymes play a role in supplementing the C4 branch of the TCA cycle by CO_2_ incorporation. Intriguingly, the *M. tuberculosis* phosphoenolpyruvate carboxykinase is under control of the same kinases that regulate the dormancy regulon [26]. However, metabolism under oxygen concentrations that are too low to sustain replication is clearly not solely controlled by the dormancy response since triacylglycerol synthase and the α-crystallin homolog HspX, both under control of the DosR dormancy response regulator, are upregulated at oxygen levels that were associated with replication and even sustained NADH/NAD^+^ ratios and ATP levels (Figure 2 and Figure 4A). Fumarate reductase has also previously been observed to be upregulated by hypoxia [25], [26], [27] a result that we confirmed by its upregulation at 0.1 mmHg dissolved oxygen. In *E. coli*, fumarate can serve as both a respiratory terminal electron acceptor as well as an electron acceptor during fermentation in the absence of preferred electron acceptors [49]. The C4 branch of the reductive TCA cycle is even utilized in human kidney cells under hypoxia. These cells anaerobically maintain electron transport and proton extrusion through the type I NADH dehydrogenase with ATP production by the mitochondrial inner membrane F_1_F_0_-ATPase [50].

Substrate feeding experiments with stable isotope labeled precursors supported the production of succinate through the reductive TCA cycle. A similar analysis of labeling patterns of succinate production in the obligate anaerobe *Clostridium acetobutylicum* also concluded that this process was dependent upon the reductive TCA cycle [51]. In this organism the TCA cycle is complete but also bifurcated so that the oxidative and reductive branches can be engaged independently of one another. Although the full details of the TCA cycle in *M. tuberculosis* remain less than completely clear, the use of intracellularly generated fumarate as an electron sink with subsequent secretion of the resulting succinic acid into the extracellular milieu suggests that, contrary to commonly accepted views, *M. tuberculosis* can maintain an energized membrane by fermentation.

The absence of a phenotype in the *frdA* knockout cells is perhaps not surprising in view of the fact that the *M. tuberculosis* genome encodes three potential fumarate reductase/succinate dehydrogenase complexes. The other two homologs may complement fumarate reductase activity under anaerobic conditions in the *frdA* knock out mutant and analysis of membrane associated fumarate/succinate dehydrogenase activities corroborates this notion since we observed similar specific enzyme activities in membrane fractions from both the *frdA* knockout and the parental wild type strain under aerobic as well as anaerobic conditions (Figure S5). The absence of a phenotype in the *frdA* knockout mutant during infection of mice is perhaps not surprising since typical strains of mice do not form the hypoxic granulomas associated with human tuberculosis disease [6]. However, the true function of fumarate reductase activity during *in vivo* pathogenesis cannot be explored with the *frdA* knockout due to the compensatory functions of the other two fumarate/succinate dehydrogenase homologs. Central metabolism such as carbon flux through the TCA cycle has been shown in other organisms to be relatively resistant to genetic modifications [52]. We also found that both malic enzyme and phosphoenolpyruvate carboxykinase were dispensable for survival under hypoxia but that genetic knockout of these enzymes resulted in compensatory upregulation of expression of the alternative enzymes that link glycolysis to the TCA cycle. These enzymes may play a key role in regulating intracellular malate/oxaloacetate pools during operation of pathways that replenish or deplete C4 metabolite levels such as the isocitrate lyase shunt, enzymes of the TCA cycle and gluconeogenesis although this function can be replaced by alternative pathways such as pyruvate carboxylase. The absence of a phenotype in the fumarate reductase, phosphoenolpyruvate carboxykinase and malic enzyme knockouts attests to the fact that the *M. tuberculosis* genome encodes considerable metabolic plasticity. However, the compensatory adaptations that occur in genetic knockout mutants have many generations to become established which would not be the case during sudden interruption of a pathway during chemical inhibition. Thus studies using genetic knockouts do not allow us to assess the functional consequences of sudden metabolic inhibition of a particular pathway that plays a critical role in a specific environment at a specific point in time.

**Figure 9:**
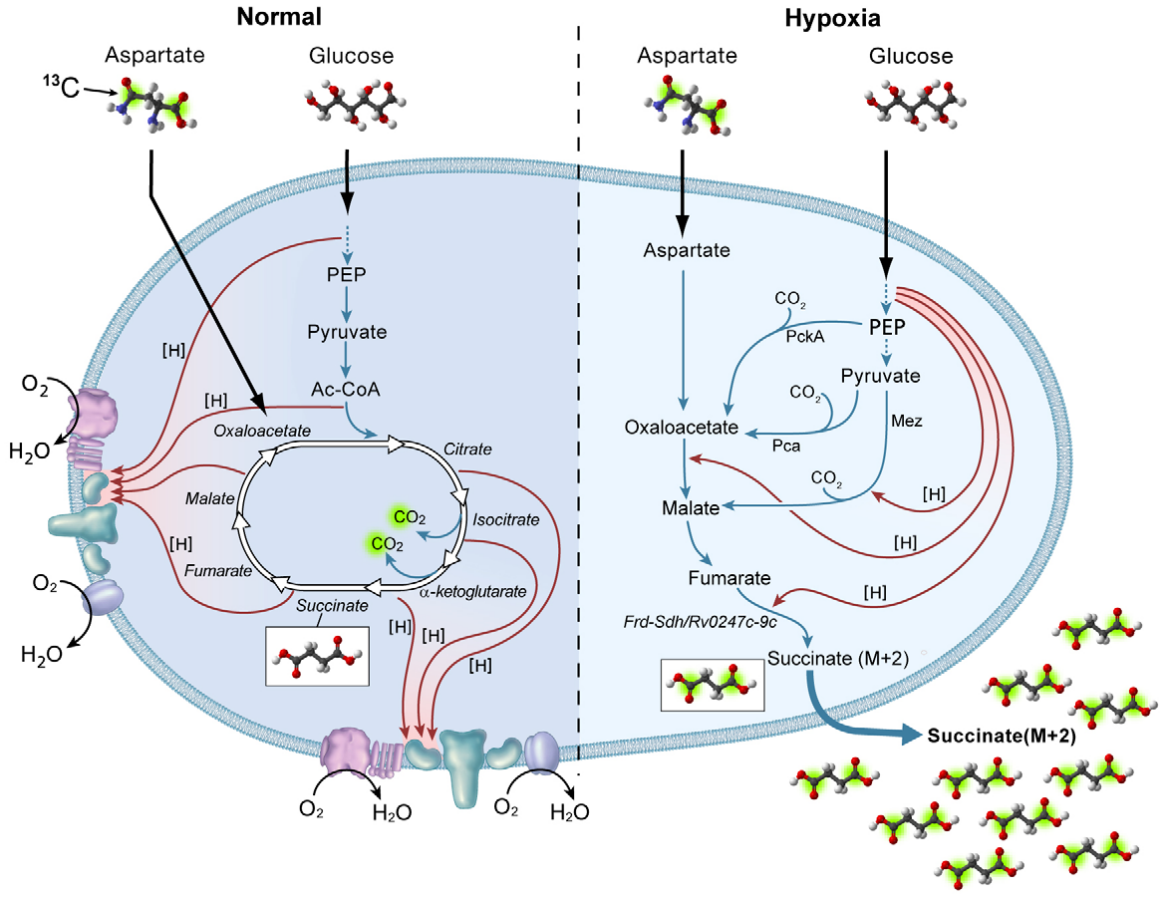
Proposed model for succinate production and NADH reoxidation in Dubos medium under anaerobic condition in *M. tuberculosis*. Under aerobic conditions, glucose is metabolized to acetyl-CoA by glycolysis and pyruvate dehydrogenase with further oxidation by the TCA cycle. Labeling of TCA cycle intermediates by feeding of cells with [1,4-^13^C_2_]-aspartate results in loss of both ^13^C atoms (green highlighting) through oxidative decarboxylation in the TCA cycle. Reduced cofactors (red arrows) are re-oxidized by respiratory complexes. Under anaerobic conditions, the direct incorporation of C3 metabolites from glycolysis into the reductive branch of the TCA cycle is favored resulting in re-oxidation of reduced cofactors. Succinate is secreted into extracellular milieu to maintain a proton motive force. Under these conditions, labeling of the C4 branch of the TCA cycle using [1,4-^13^C_2_]-aspartate results in secretion of succinate labeled at the C1 and C4 positions with ^13^C.

The metabolic pathways utilized by *M. tuberculosis* under anaerobic conditions will also depend on substrate availability. In our experiments *M. tuberculosis* was grown in Dubos medium which contains glucose as well as asparagine, glucose is metabolized by glycolysis to yield ATP by substrate level phosphorylation as well as reduced cofactors. Restriction of oxygen limits the oxidation of glucose in the oxidative direction of the TCA cycle with the reductive branch taking precedence in the absence of oxygen (Figure 9). The intermediates in the reductive branch would be further supplemented by the excess asparagine in Dubos medium which can be metabolized to aspartate and by further transamination to oxaloacetate. We observed a slight increase in lactic acid secretion in our anaerobic cultures over time (Figure 3A). Fermentation by lactic acid secretion would provide another means of membrane energization and NADH reoxidation although in our experimental set-up fumarate reduction was favored. Lactic acid production through lactic acid dehydrogenase may play a more prominent role under other growth conditions. However, even in growth media where the nitrogen source was L-alanine, succinate accumulation was observed despite the formation of substantial levels of pyruvate, suggesting that fumarate reduction is preferred under a variety of nutrient conditions. The genome encodes several other potential respiratory enzymes of unknown function, some of which are upregulated during hypoxia in a DosR dependent manner [13], [29], that may also play a role in maintenance of an energized membrane under anaerobic conditions.

We therefore conclude that fermentation plays a critical role in maintaining an energized membrane under conditions where there is restricted availability of oxygen. Irrespective of the available carbon source, oxidation of such substrates through glycolysis or through oxidation of substrates such as cholesterol or lipids, would lead to accumulation reduced cofactors. In the complete absence of an external electron acceptor, oxidation of cholesterol or fatty acids would not lead to ATP generation since metabolism of these substrates does not produce enough high energy intermediates through substrate level phosphorylation. However, the granuloma, though hypoxic, is not completely anaerobic and limited availability of oxygen would allow limited metabolism of such substrates with concomitant oxidative phosphorylation. Reduction of fumarate reoxidizes more reducing equivalents than are produced during glycolysis which could favor co-metabolism of several carbon sources. Replenishment of the C4 branch of the TCA cycle would also be possible by metabolism of substrates such as aspartate which directly contribute to the C4 branch of the cycle or possibly even by CO_2_ fixation of glycolytic intermediates or the pyruvate produced by the methylisocitrate cycle during metabolism on substrates such as cholesterol, the breakdown of which yields propionyl-CoA (as well as pyruvate and acetyl-CoA). The notion that glycolytic intermediates feed into the reductive branch of the TCA cycle by a reverse gluconeogenic processes is particularly appealing considering the environment of the lung where CO_2_ concentrations are more than 10-fold higher than atmospheric CO_2_ concentrations [53]. The presence of several potential enzymes that can reduce fumarate poses a dilemma for the purpose of inhibitor development to explore the essentiality of pathways under *in vivo* relevant conditions. However, the reverse branch of the TCA cycle contains other potentially vulnerable points, particularly the non-redundant fumarase [54]. These studies show that fermentation plays an important role in the maintenance of tuberculosis infection under oxygen tensions proven relevant in granulomatous infections in higher vertebrates, a better understanding of this process may lead to the rational selection of targets for latent infection as well as holding the potential for shortening the duration of tuberculosis chemotherapy.

## Materials and Methods

### *M. tuberculosis* growth conditions

*M. tuberculosis* strain H_37_Rv and Erdman and their mutant strains H_37_Rv Δ*mez*, H_37_Rv Δ*frdA*, Erdman Δ*icl1*/Δ*icl2* and Erdman Δ*pckA* mutant strains and *pckA* complemented strain (101) were grown in Dubos medium (Becton Dickinson; as supplied supplemented with Tween 80) supplemented with albumin/dextrose/NaCl (ADC) enrichment. 50 µg/ml hygromycin or 50 µg/ml kanamycin were used for Δ*mez*, Δ*icl1/*Δ*icl2*, Δ*pckA* or Δ*frdA* cultures, respectively. Non-replicating persistent (NRP) cells were obtained by subjecting bacterial cultures to slow magnetic stirring in sealed tubes with a head/space ratio of 0.5 as described [22].

To determine the growth rate under various oxygen concentrations, H_37_Rv was incubated in a 1 l Biostat A-plus fermentor (Sartorius North America). Dissolved oxygen concentration was measured by InPro 6800 D.O. sensor (Mettler Toledo). The sensor was calibrated by helium gas for zero point and air gas for slope. We also confirmed the sensitivity of the sensor by a fiber optic oxygen sensor (OxyLite 4000; Oxford Optronix, Oxford, United Kingdom) and enzymatic oxygen measurement [55].

8.5 ml of preculture (OD_650 nm_ = 0.25) of *M. tuberculosis* H_37_Rv was inoculated into 850 ml of Dubos media in fermentors and incubated at 37°C with slow stirring (50 rpm) without gas feeding for 3 days. Dissolved oxygen tensions were gradually decreased over the next three days by feeding a mixture of air and 100% nitrogen. The cultures reached each set point of oxygen concentration at day 6 after which the oxygen concentration was maintained at the final desired value. Culture pH was controlled at 6.5 by 1 N HCl or 1 N NaOH. Growth rate and doubling times were calculated based on the absorbance and colony forming units (CFU) which were measured daily for 11 days after reaching the set oxygen concentration. For continuous cultures, *M. tuberculosis* H_37_Rv was grown in fermentors under defined dissolved oxygen tensions, 50.0 mmHg, 8.16 mmHg, 1.85 mmHg, 1.44 mmHg and 0.42 mmHg. 90 ml of preculture (OD_650_ = 0.2–0.3) were inoculated into 900 ml of Dubos media in the fermentors and incubated at 37°C with slow stirring (50 rpm) without gas feeding for 3 days after which air and nitrogen gas were fed to achieve the final desired oxygen concentration over the 24 h. Continuous culture also started on day 3 with a constant growth rate of 0.0077 h^−1^ with culture volume maintained at 900 ml and reached steady state on day 26 based on culture absorbance, biomass and CFU/ml. Chemostat cultures were harvested on day 33 and 34. To incubate *M. tuberculosis* under various oxygen tensions with 5% CO_2_, nitrogen and air gas balanced with 5% CO_2_ gas were used instead of air and nitrogen gas.

### Generation of mutant strains

The *frdA* of the fumarate reductase operon and *mez* genes were inactivated by allelic replacement. A 4897 bp *Hind* III cosmid DNA fragment containing 1909 bp of upstream sequence from the *frdA* start codon was cloned into pGEM3Zf(+). The *aph* gene was cloned as a *Pst* I blunt-ended fragment into the *Eco*RV site of *frdA* which creates an insertional inactivation 1089 bp into the *frdA* gene hereby inactivating the downstream dehydrogenase (pfam02910) domain. The *Pac* I fragment containing the *sacB* and *lacZ* genes from pGOAL17 [56] was cloned into the *Sca* I site of this plasmid to generate pGfrdAKO which was used for electroporation of *M. tuberculosis*. For *mez*, a 5669 bp *Kpn* I – *Eco* RV fragment containing 1086 bp and 2936 bp upstream and downstream of *mez*, respectively, was cloned into pcDNA2.1. The *mez* gene was disrupted by insertional activation by cloning a hygromycin resistance cassette halfway into the gene in the *Bgl* II site. A *Pac* I fragment containing the *sacB* and *lacZ* genes from pGOAL17 was cloned into the *Eco*R V site of this plasmid to generate pcmezKO which was used for electroporation of *M. tuberculosis*. Electroporation and generation of double crossover strains was performed as previously described [25], [56].

### LC-MS analysis

^13^C –labeled Succinate and non-labeled organic acids (pyruvate, malate, succinate, lactate and fumarate) were measured by LC/MS as described [32]. Liquid chromatography was performed on an Agilent 1100series LC/MS system (Wilmington, DE, USA) with a diode array UV/Vis detector (DAD, model G1315A) and a single quadrupole mass-selective detector (MSD, model G1946DSL). Separation was carried out using an ion exclusion column (Aminex, Bio-Rad) HPX-87H, 7.8 mm-300 mm, with guard column, heated to 40°C, with an isocratic flow rate of 0.8 ml/min of 0.1% (v/v) formic acid in water. The injection volume was 20 µl for all samples. System control and data analysis were performed using Agilent Chemstation version A.09.03. The MSD used electrospray ionization in negative ion mode with selective ion monitoring (SIMES-). Ions investigated were 117, 118, 119, 120, 121 for succinate anion.

All water used was delivered from a Barnstead Diamond nanowater purifier with greater than 18.1 MΩ-cm resistivity, succinic acid (≥99%) standards were purchased from Sigma-Aldrich (St. Louis, MO, USA). All mobile phases were used without pH adjustment.

### Whole cell labeling with ^13^C-labeled substrates

NRP cultures and aerobically growing cultures were labeled with [1, 4-^13^C_2_] L-aspartic acid (99%), [U-^13^C| L-aspartic acid (98%), [U-^13^C] D-glucose (99%) (Cambridge Isotope Laboratories, Inc.) or ^13^C Sodium bicarbonate (98%) (Isotec). 187.5 µl of 20 mg/ml ^13^C-sources were added into 1.5 ml of aerobic culture (OD_650 nm_ = 0.17) or NRP culture with substrate addition to anaerobic cultures performed in an anaerobic chamber (MACS MG 1000 Anaerobic workstations, Don Whitley Scientific). After 24 h incubation at 37°C, culture supernatants were harvested by filtration through 0.22 µm membranes, and frozen at −20°C prior to LC-MS analysis.

In order to follow the time course of isotopic succinate accumulation in the supernatant of NRP culture in Dubos medium with [U-^13^C]glucose, H_37_Rv was incubated in 10 ml of Dubos medium where Glucose was replaced to [U-^13^C]glucose in Wayne model tube with septum. 1 ml of the culture supernatant was collected every 5 days and analyzed by LC-MS.

For metabolomics analyses of aerobic cells, *M. tuberculosis* was grown in Dubos medium supplemented with ADC enrichment. At an OD_650 nm_ = 0.25, cells were harvested and washed in Dubos medium supplemented with Albumin/NaCl but without glucose (carbon source-free Dubos medium). Cells were resuspended in carbon source-free Dubos medium OD_650 nm_ = 0.9 and supplemented with 7.5 mg/mL U-^13^C-glucose and returned to the 37°C with shaking. At various time points, 2 mL aliquots were rapidly harvested through 0.8 µm filters by vacuum filtration and washed with saline with the entire harvesting and washing procedure taking less than 20 s. Filters were immediately submerged in 2∶1 chloroform:methanol at −80°C overnight after which the solvent was evaporated at room temperature *in vacuo*. The metabolites were resuspended in 3×1 mL 60% (v/v) ethanol and pooled fractions were dried at 30°C in a SpeedVac equipped with a cooling trap at −85°C. The dried extracts were dissolved in 100 µL 5% (v/v) methanol in water for metabolite analysis by LC-MS/MS [57]. Isotopomer distributions were quantified as described in (Rühl et al, in press). For LC-MS/MS analysis of secreted succinate, culture supernatant was directly tenfold diluted in 5% (v/v) methanol and stored at −80°C until injection. For metabolomic analyses of anaerobically adapted cells, *M. tuberculosis* was grown into non-replicating persistence as described (Wayne & Hayes, 1996). After 2 and 3 weeks of hypoxic adaptation, cells were anaerobically harvested, washed in anaerobic, carbon source-free Dubos medium, resuspended to an OD_650 nm_ of 1.0 in carbon source-free Dubos medium supplemented with 7.5 mg/mL [U-^13^C]glucose and maintained at 37°C in the anaerobic chamber. At various time points, cells were harvested, washed and metabolites prepared as described above.

### Measurement of NAD(H) and ATP

NAD and NADH concentrations were measured by rapidly harvesting (60 sec in microfuge) 1 ml cultures which were frozen on dry ice and cofactor determination by the NAD/NADH recycling assay (San 2002 Metab Eng 4, 182–192 and Boshoff 2004 JBC 279, 40174–40184). For determination of the corresponding dry cell weight, 50 ml of culture was harvested, resupended in water, transferred into a pre-weighed tube and dried to constant weight at 80°C. ATP levels were determined by BacTiter-Glo Microbial Cell Viability Assay (Promega).

### RNA Isolation and qRT-PCR

RNA was prepared as described previously [58]. First-strand cDNA synthesis was performed by Super Script III First-Strand Synthesis Super Mix for qRT-PCR (Invitrogen). Real-time quantitative PCR was carried out on the ABI Prism 7700 sequence detection system with iTaq Supermix With ROX (Bio Rad) using Molecular Beacons system [59]. The primers and probes are described in Table S2. The thermal cycling conditions were initial denaturation at 95°C for 3 min followed by 40 PCR cycles with denatureation at 95°C for 30 sec, annealing at 54°C for 1 min, and extension at 72°C for 30 sec. Fluorescence measurements were recorded at each annealing step.

### Succinate dehydrogenase assay

*M. tuberculosis* H_37_Rv and Δ*frdA* cells were pelleted and washed with pre-cold 10 mM potassium phosphate buffer (pH 7.0) plus 1 mM DL-dithiothreitol. The cells were dissolved in 10 mM potassium phosphate buffer with 1 mM DTT and lysed by beat beading. The suspension was centrifuged at 12,000 rpm for 10 min at 4°C. The supernatant was further centrifuged at 200,000×*g* for 2 h at 4°C. The pellet was suspended in 50 mM potassium phosphate buffer (pH 7.0) containing 1 mM 2-mercaptoethanol and 1 mM MgCl_2_. Protein concentration of the membrane fraction was determined by Coomassie Protein Assay Reagent (Thermo Scientific). The succinate dehydrogenase activity was measured according to the method of Munujos [60].

### *In vivo* experiments

Strains were grown to an OD_650 nm_ of 0.2, filtered through a 5 µm filter and subsequently diluted into phosphate buffered saline supplemented with 0.05% Tween 80. Eight-week-old C57Bl/6 mice were infected with 100 colony forming units of the *M. tuberculosis* suspensions by aerosol using a BioAerosol nebulizing generator (CH Technologies Inc., Westwood, NJ) for 10 min. Groups of 5 mice for each *M. tuberculosis* strain were euthanized at the indicated time points and lungs and spleens homogenized in 7H9 Middlebrook supplemented medium and appropriate dilutions plated on 7H11 Middlebrook agar plates supplemented with 10% OADC.

### Ethics statement

All animal experiments were conducted in accordance with the animal care and use committee of NIAID DIR, under animal study protocol LCID-3E. The Animal Care and Use Committee (ACUC) of the National Institute of Allergy and Infectious Diseases, Division of Intramural Research, with permit number NIH IRP, PHS Assurance A4149-01, approved the animal study protocol LCID-3E under which all animal experiments were performed.

### Accession numbers

Gene name: UniProt accession number

frdA: P64174; frdB: Q10761; frdC: Q10762; frdD: P67643; sdhA: O53370; sdhB: O53371; sdhC: O53368; sdhD: O53369; Rv0247c: O53669; Rv0248c: O53670; Rv0249c: O53671; mez: P71880; pca: P95127; pckA: P65686; tgs: P0A650; hspX: P0A5B7; citA: P63777; acn: O53166; kgd: O50463

## Supporting Information

Figure S1 **Transcriptional profile of genes related to central carbon metabolism under various dissolved oxygen tensions.** Gene expression levels of 8, 2, 1, 0.5 and 0.1 mmHg culture grown with or without 5% carbon dioxide were presented as ratios compared with 50 mmHg culture; (A) *frdA*, (B)*sdhA*, (C) *Rv0248c*, (D) *mez*, (E), *pckA*, (F) *pca*, (G) *aceE*, (H) *pdhA*, (l) *lldD1*, (J) *citA*, (K) *acn*, (L) *icd1*, (M) *icd2*, (N)*kgd*, (O) *korA*, (P) *fum*, (Q) *icl1*, (R) *ilvB2*, (S) *hsaG*, (T) *accA1*, (U) *glpD1*, (V) *Rv3837c*, (W) *tgs1*, and (X) *hspX*. They were normalized to the expression levels of *sigA*.
(DOC)

Figure S2 **Transcriptional profile of genes related to central carbon metabolism under six different oxygen concentrations.** Schematic illustration of carbon metabolic pathways and heat map of expression profile for *M. tuberculosis* grown under 6 different oxygen concentrations. Gene expression levels of 8, 2, 1, 0.5 and 0.1 mmHg culture grown with or without 5% carbon dioxide were presented as ratios compared with 50 mmHg culture. They were normalized to the expression levels of *sigA*. The grid inset shows the corresponding dissolved oxygen tensions shown for the heatmap of fold expression changes relative to 50 mmHg dissolved oxygen tension (DOT). The color scale inset shows the corresponding coloring for fold expression changes of each gene at the DOT under investigation relative to 50 mmHg.
(TIF)

Figure S3 **Transcriptional profile of select central metabolic genes in gene knockout mutants under six different oxygen concentrations.** Complement upregulation of alternative pathway in *frdA*, *mez* and *pckA* knock out mutants. Expression levels of 15 days NRP cultures were expressed as ratios compared with aerobic culture and normalized to the levels of *sigA*. Analysis of (A) succinate dehydrogenase genes in the *frdA* mutant and its H37Rv parental wild type strain, (B) genes connecting glycolysis to the C4 branch of the TCA in the *mez* mutant and H37Rv parental wild type strain, (C) genes connecting glycolysis to the C4 branch of the TCA in the *pckA* mutant and Erdman parental wild type strain.
(TIFF)

Figure S4 **Intracellular and extracellular isotopomer distribution of metabolites of the C4 branch of the TCA cycle of NRP cultures in U-^13^C Glucose Dubos medium.** H_37_Rv was adapted to hypoxia in the Wayne model using Dubos medium where the glucose had been replaced with U- ^13^C glucose. Intracellular and extracellular metabolites were analyzed at the indicated time points. Only significant levels of succinate could be detected in the extracellular medium.
(TIF)

Figure S5 **Membrane associated succinate dehydrogenase activities of wild-type or fumarate reductase knockout strains adapted to aerobic or anaerobic conditions.** Membrane fractions were prepared from H_37_Rv and H_37_Rv Δ*frdA* grown aerobically in Dubos medium or adapted to anaerobic conditions in Wayne model tubes followed by measurement of succinate dehydrogenase activities.
(TIFF)

Figure S6 **Growth and survival of wild-type or Δ** ***frdA*** **mutant** ***in vivo*** **.** Lungs C57Bl/6 mice were infected with 100 CFU of the wild-type and Δ*frdA* strains followed by monitoring of bacterial burdens in (A) lungs and (B) spleens in mice over time. Each time point represents the median CFU and standard error of 5 mice per group.
(TIFF)

Table S1 **Organic acid accumulation in the supernatant of** ***M. tuberculosis*** **culture.**
(XLS)

Table S2 **Oligonucleotide sequences of primers and probes used for qRT-PCR.**
(XLS)
