# Supplementary material for: Fumarate Reductase Activity Maintains an Energized Membrane in Anaerobic Mycobacterium tuberculosis
Source: PLoS Pathog. 2011 Oct 6;7(10):e1002287. doi: 10.1371/journal.ppat.1002287 (PMC3188519; doi:10.1371/journal.ppat.1002287)

**Figure S1 Transcriptional profile of genes related to central carbon metabolism under various dissolved oxygen tensions.**

Gene expression levels of 8, 2, 1, 0.5 and 0.1 mm Hg culture grown with or without 5% carbon dioxide were presented as ratios compared with 50 mm Hg culture. They were normalized to the expression levels of *sigA*. RNAs were extracted from continuous cultures which were incubated with constant growth rate of 0.0077 h^-1^ under 50 to 0.5 mm Hg dissolved oxygen tensions. RNA was also extracted from batch culture where oxygen tension was being kept under 0.1 mm Hg for 10 days.


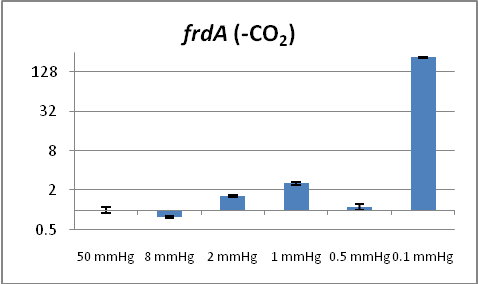
A. *frdA* (Rv1552)


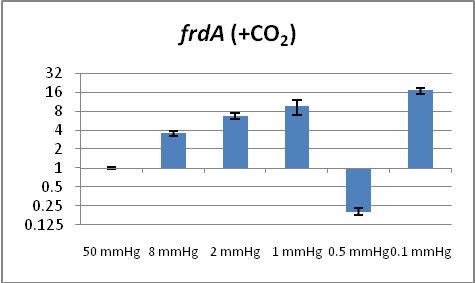


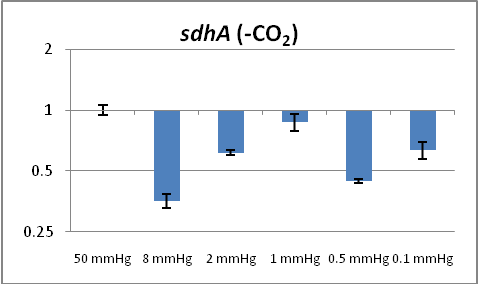
B. *sdhA* (Rv3318)


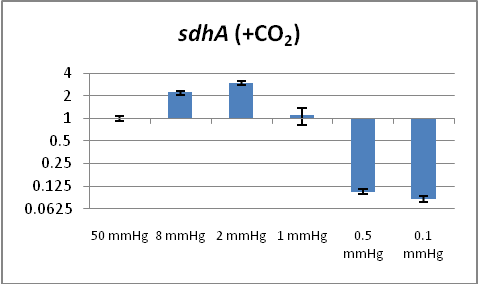


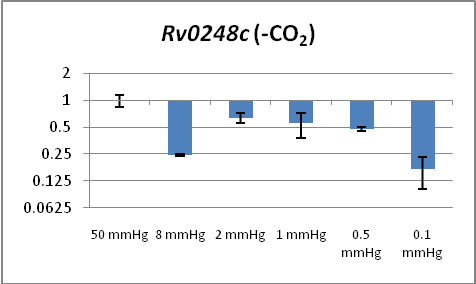
C. *Rv0248c*


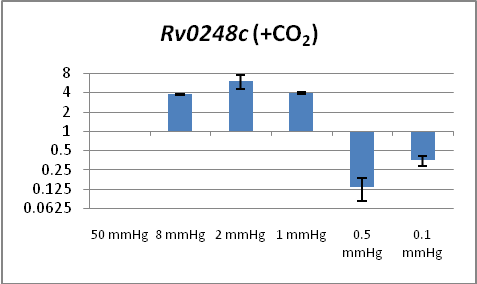


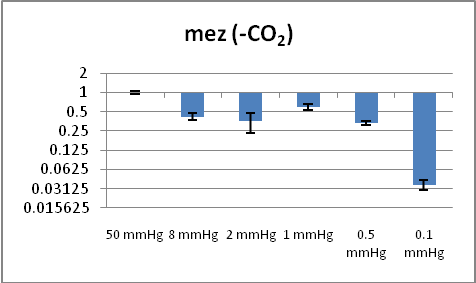
D. *mez* (Rv2332)


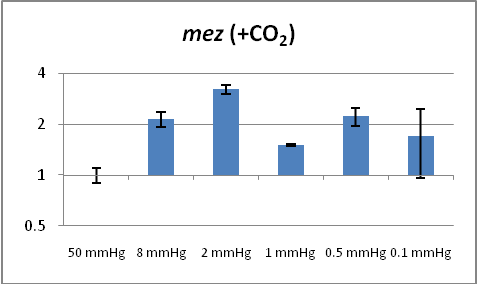


E. *pckA* (Rv0211)


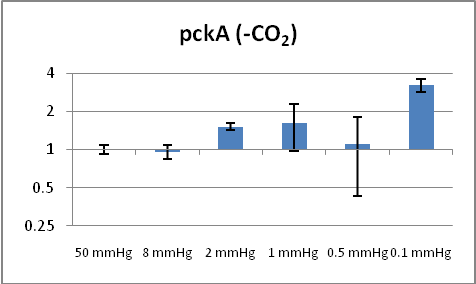

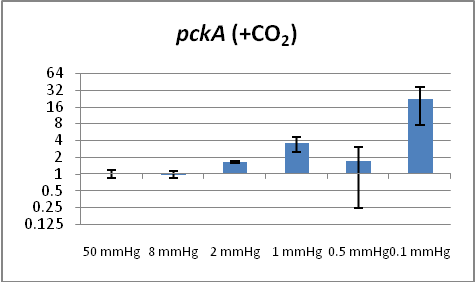


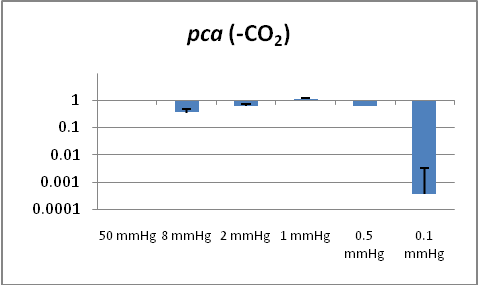
F. *pca* (Rv2967c)


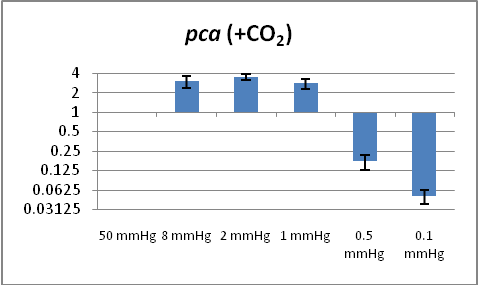


G. *aceE* (Rv2241)


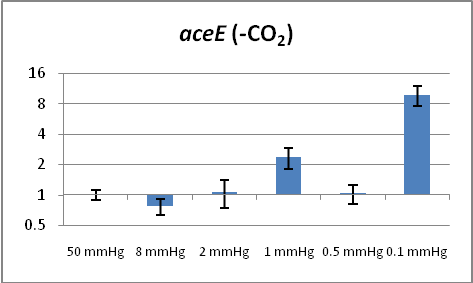

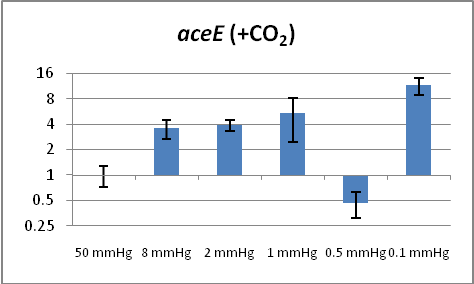


H. *pdhA* (Rv2497c)


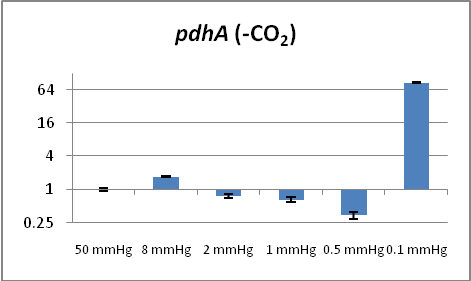

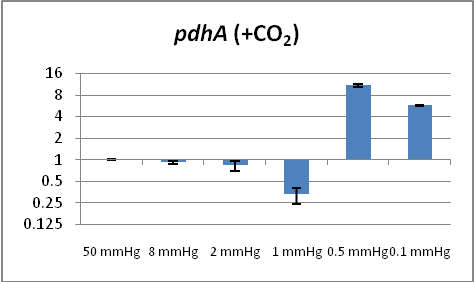


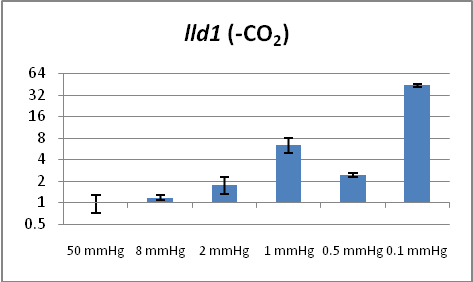
I. *lldD1* (Rv0694)


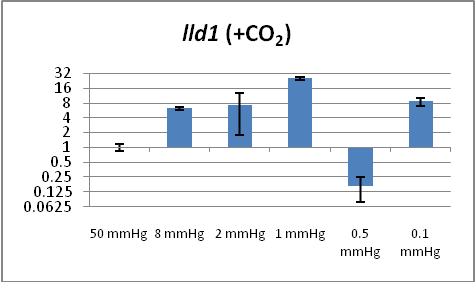


J. *citA* (Rv0889c)


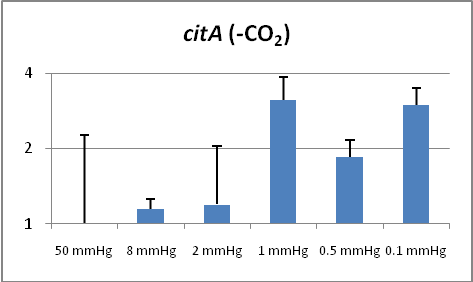

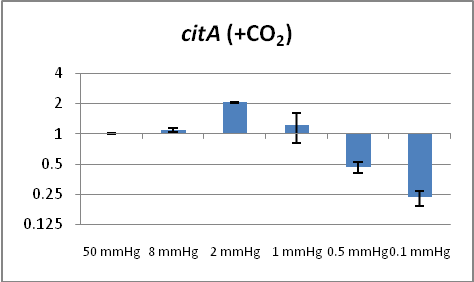


K. *acn* (Rv1475c)


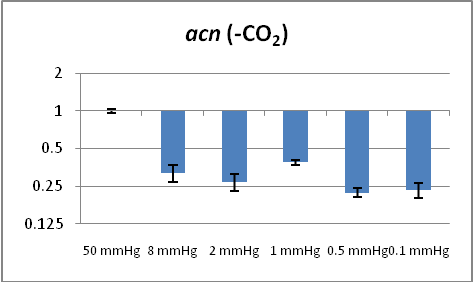

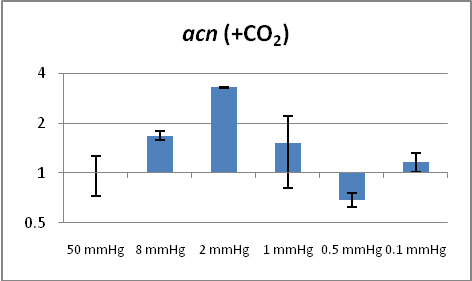


L. *icd1* (Rv3339c)


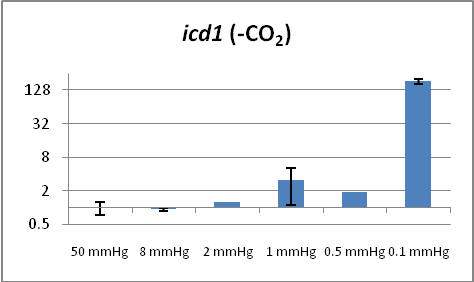

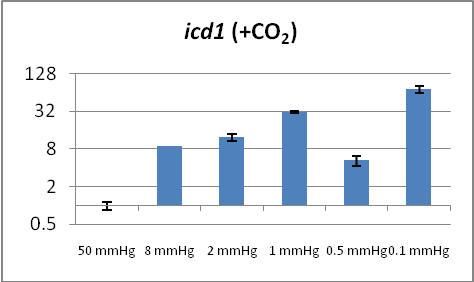


M. *icd2* (Rv0066c)


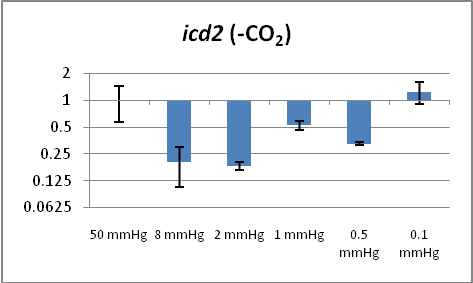

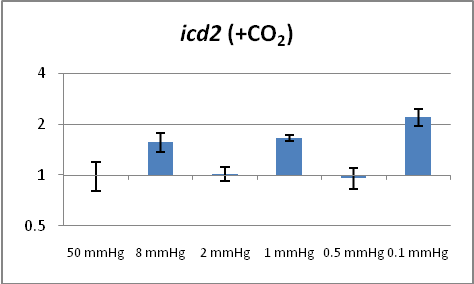


N. *kgd* (Rv1248c)


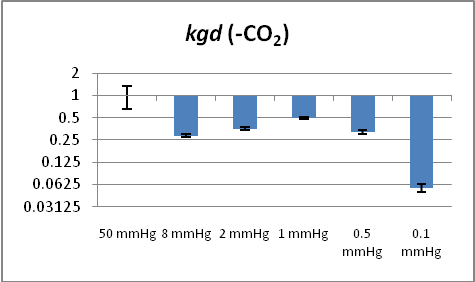

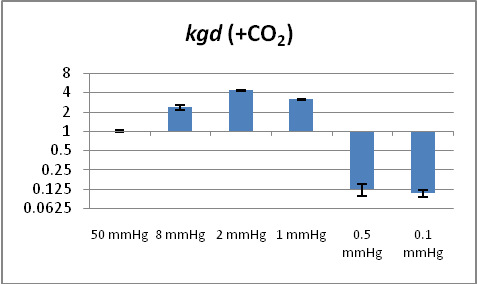


O. *korA* (Rv2455c)


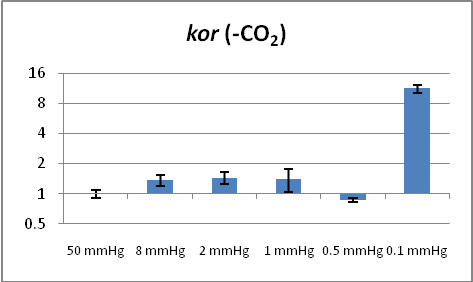

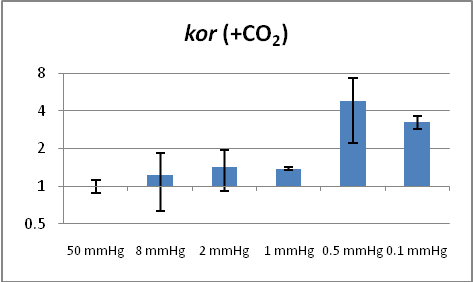


P. *fum* (Rv1098c)


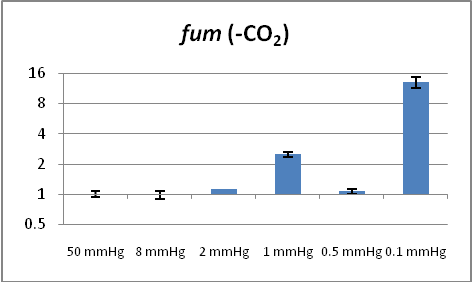

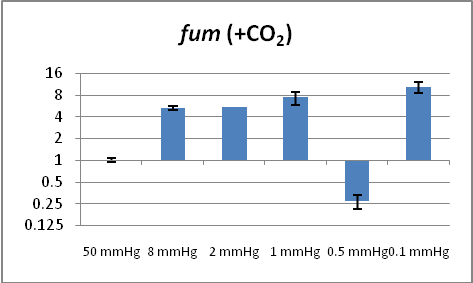


Q. *icl1* (Rv0467)


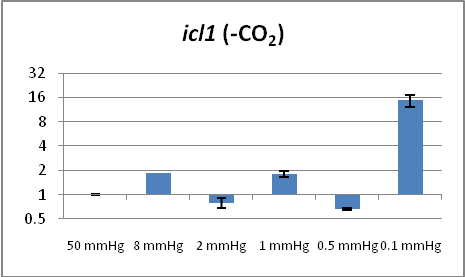

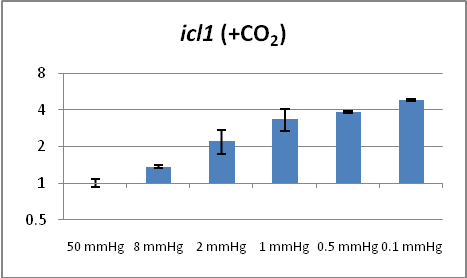


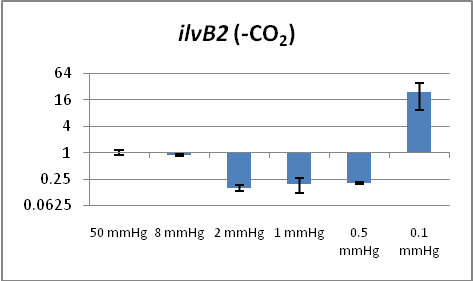
R. *ilvB2* (Rv3470c)


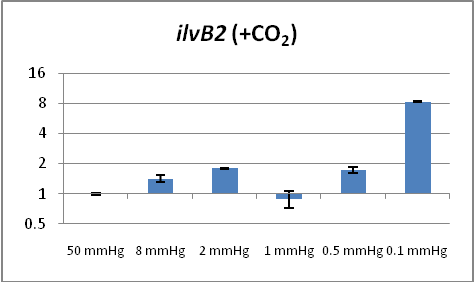


S. *hsaG* (Rv3535c)


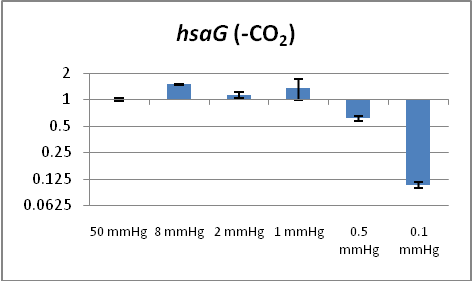

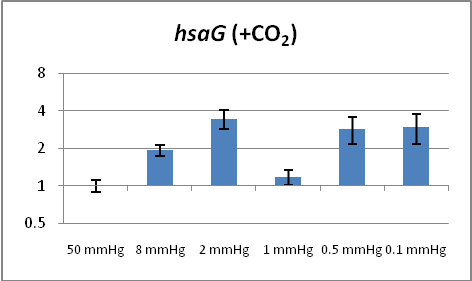


T. *accA1* (Rv2501c)


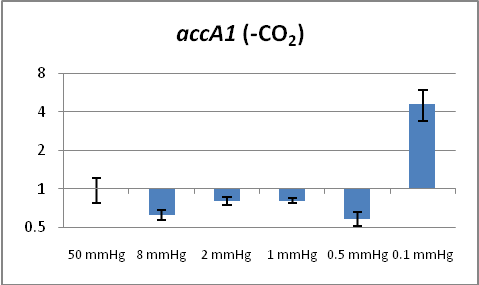

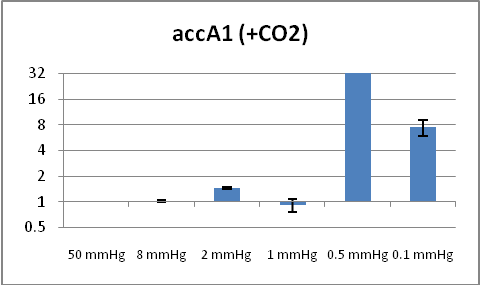


U. *glpD1* (Rv2249)


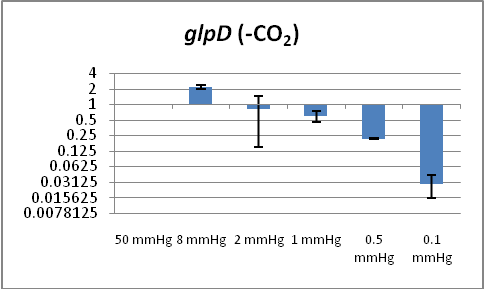

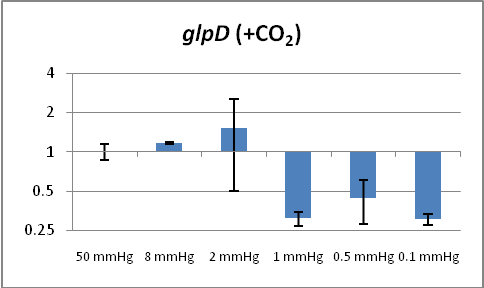


V. Rv3837c


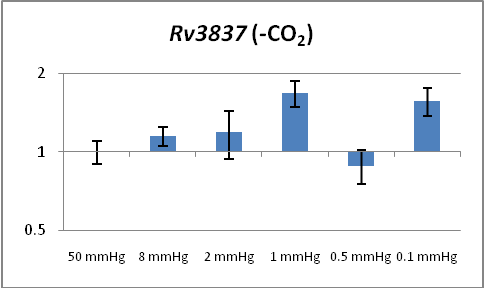

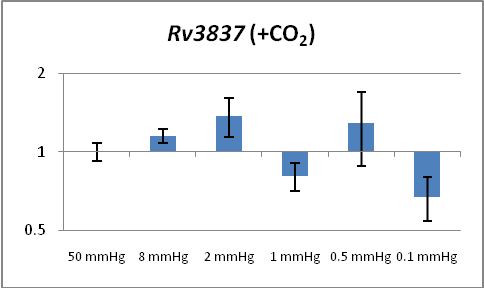


W. *tgs1* (Rv3130c)


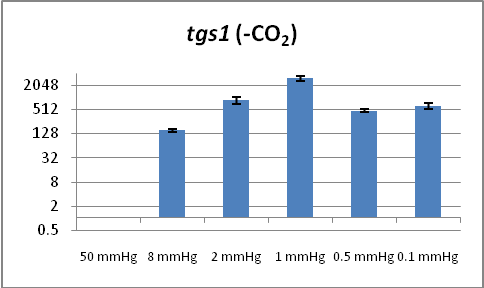

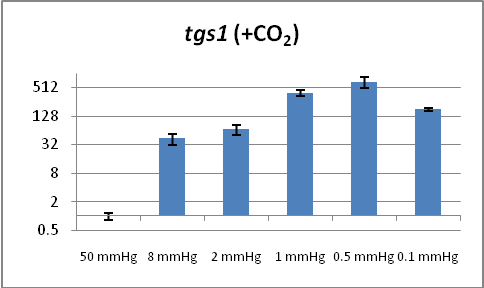


X. *hspX* (Rv2031c)


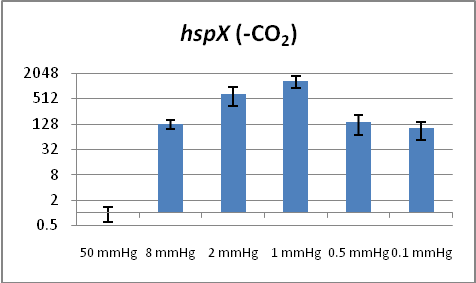

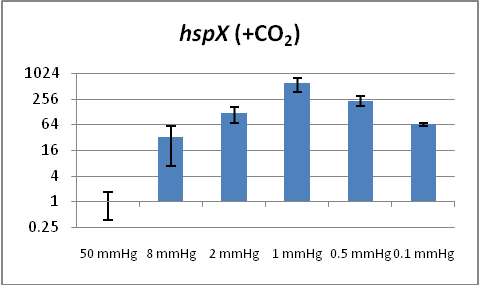

Supplement: Figure S1 — Transcriptional profile of genes related to central carbon metabolism under various dissolved oxygen tensions. Gene expression levels of 8, 2, 1, 0.5 and 0.1 mmHg culture grown with or without 5% carbon dioxide were presented as ratios compared with 50 mmHg culture; (A) frdA, (B)sdhA, (C) Rv0248c, (D) mez, (E), pckA, (F) pca, (G) aceE, (H) pdhA, (l) lldD1, (J) citA, (K) acn, (L) icd1, (M) icd2, (N)kgd, (O) korA, (P) fum, (Q) icl1, (R) ilvB2, (S) hsaG, (T) accA1, (U) glpD1, (V) Rv3837c, (W) tgs1, and (X) hspX. They were normalized to the expression levels of sigA. (DOC) [file ppat.1002287.s001.doc]
