# Supplementary figures and images for: Fumarate Reductase Activity Maintains an Energized Membrane in Anaerobic Mycobacterium tuberculosis
Source: PLoS Pathog. 2011 Oct 6;7(10):e1002287. doi: 10.1371/journal.ppat.1002287 (PMC3188519; doi:10.1371/journal.ppat.1002287)

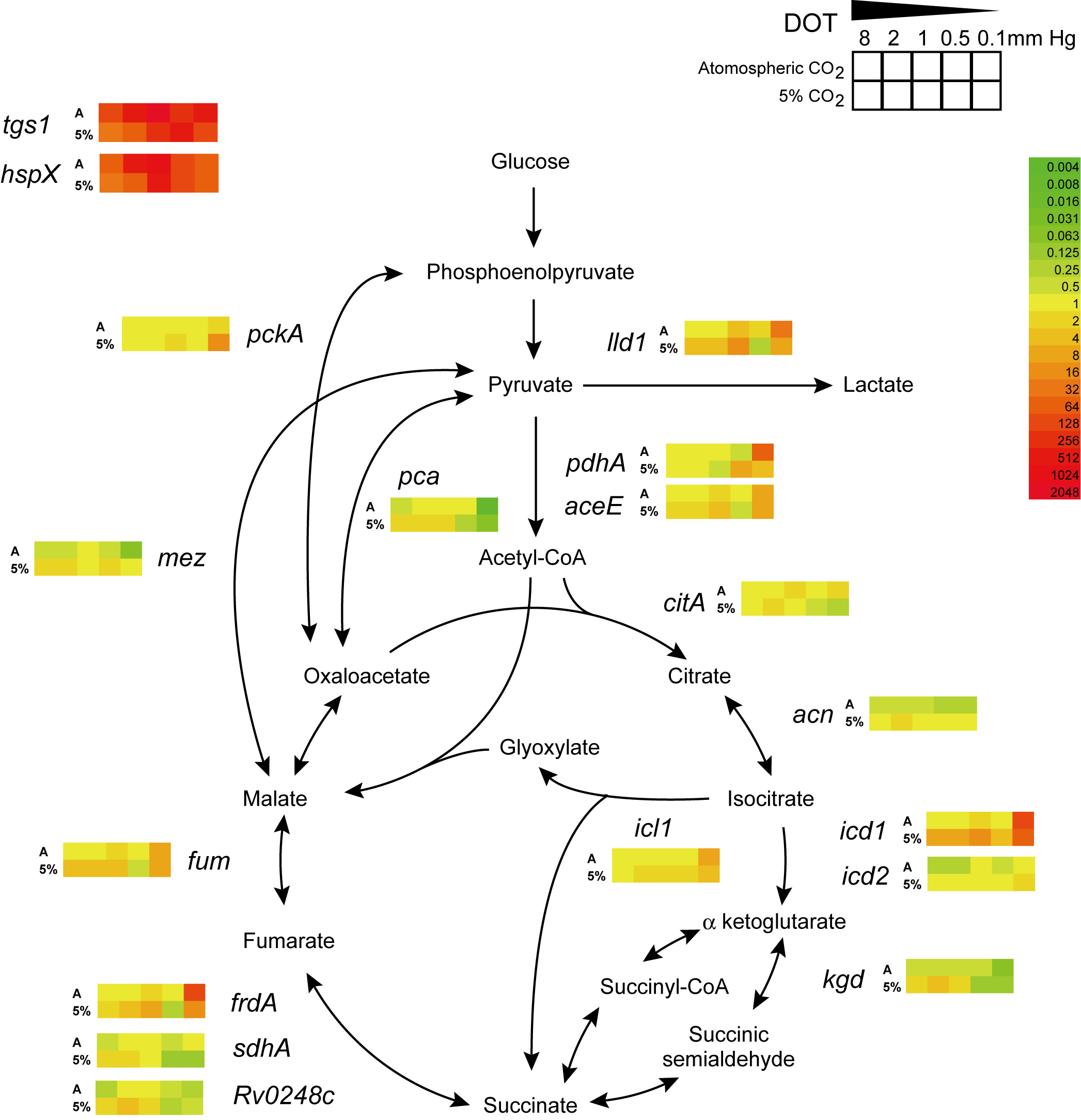

Supplement: Figure S2 — Transcriptional profile of genes related to central carbon metabolism under six different oxygen concentrations. Schematic illustration of carbon metabolic pathways and heat map of expression profile for M. tuberculosis grown under 6 different oxygen concentrations. Gene expression levels of 8, 2, 1, 0.5 and 0.1 mmHg culture grown with or without 5% carbon dioxide were presented as ratios compared with 50 mmHg culture. They were normalized to the expression levels of sigA. The grid inset shows the corresponding dissolved oxygen tensions shown for the heatmap of fold expression changes relative to 50 mmHg dissolved oxygen tension (DOT). The color scale inset shows the corresponding coloring for fold expression changes of each gene at the DOT under investigation relative to 50 mmHg. (TIF) [file ppat.1002287.s002.tif]

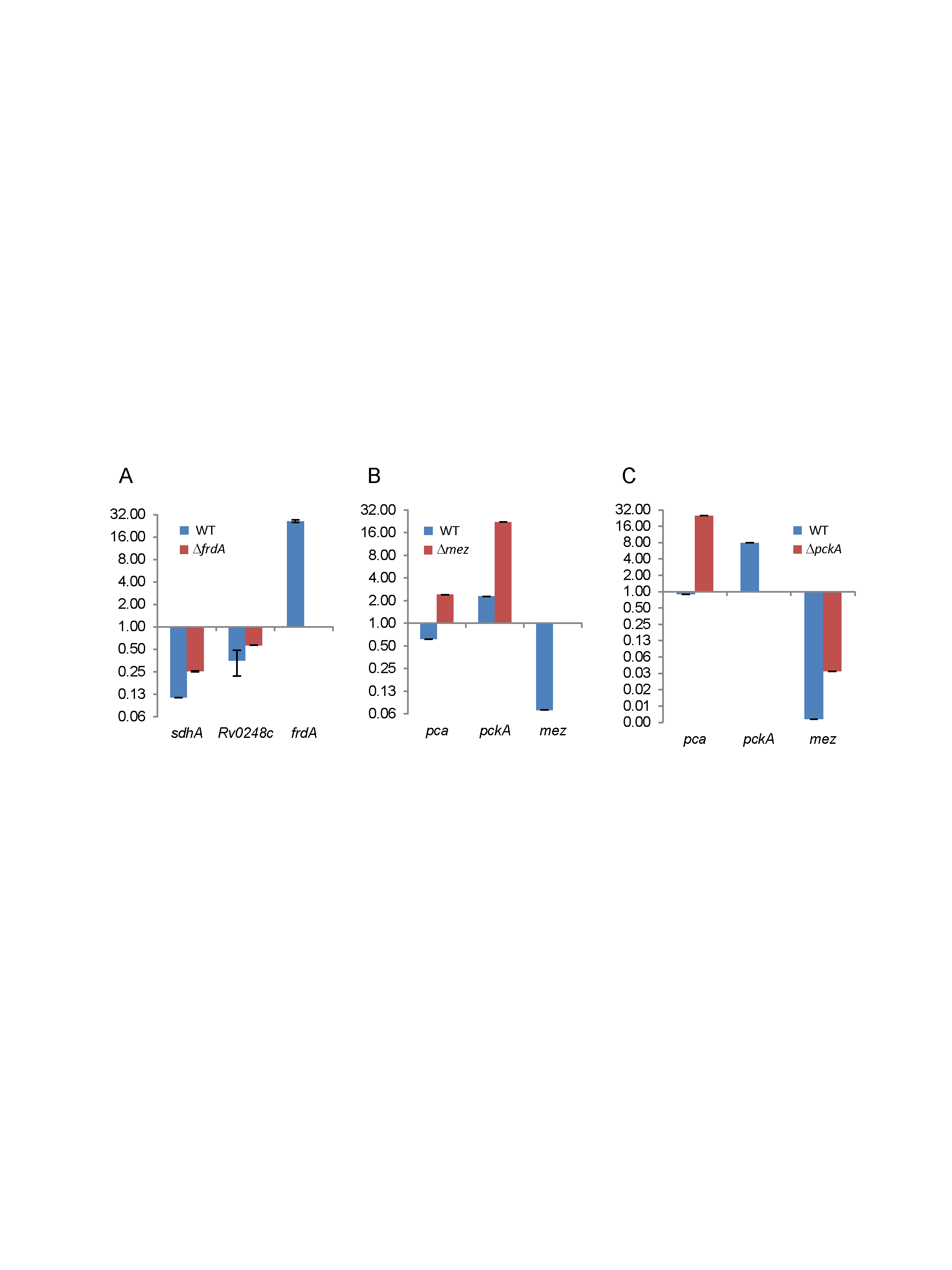

Supplement: Figure S3 — Transcriptional profile of select central metabolic genes in gene knockout mutants under six different oxygen concentrations. Complement upregulation of alternative pathway in frdA, mez and pckA knock out mutants. Expression levels of 15 days NRP cultures were expressed as ratios compared with aerobic culture and normalized to the levels of sigA. Analysis of (A) succinate dehydrogenase genes in the frdA mutant and its H37Rv parental wild type strain, (B) genes connecting glycolysis to the C4 branch of the TCA in the mez mutant and H37Rv parental wild type strain, (C) genes connecting glycolysis to the C4 branch of the TCA in the pckA mutant and Erdman parental wild type strain. (TIFF) [file ppat.1002287.s003.tiff]

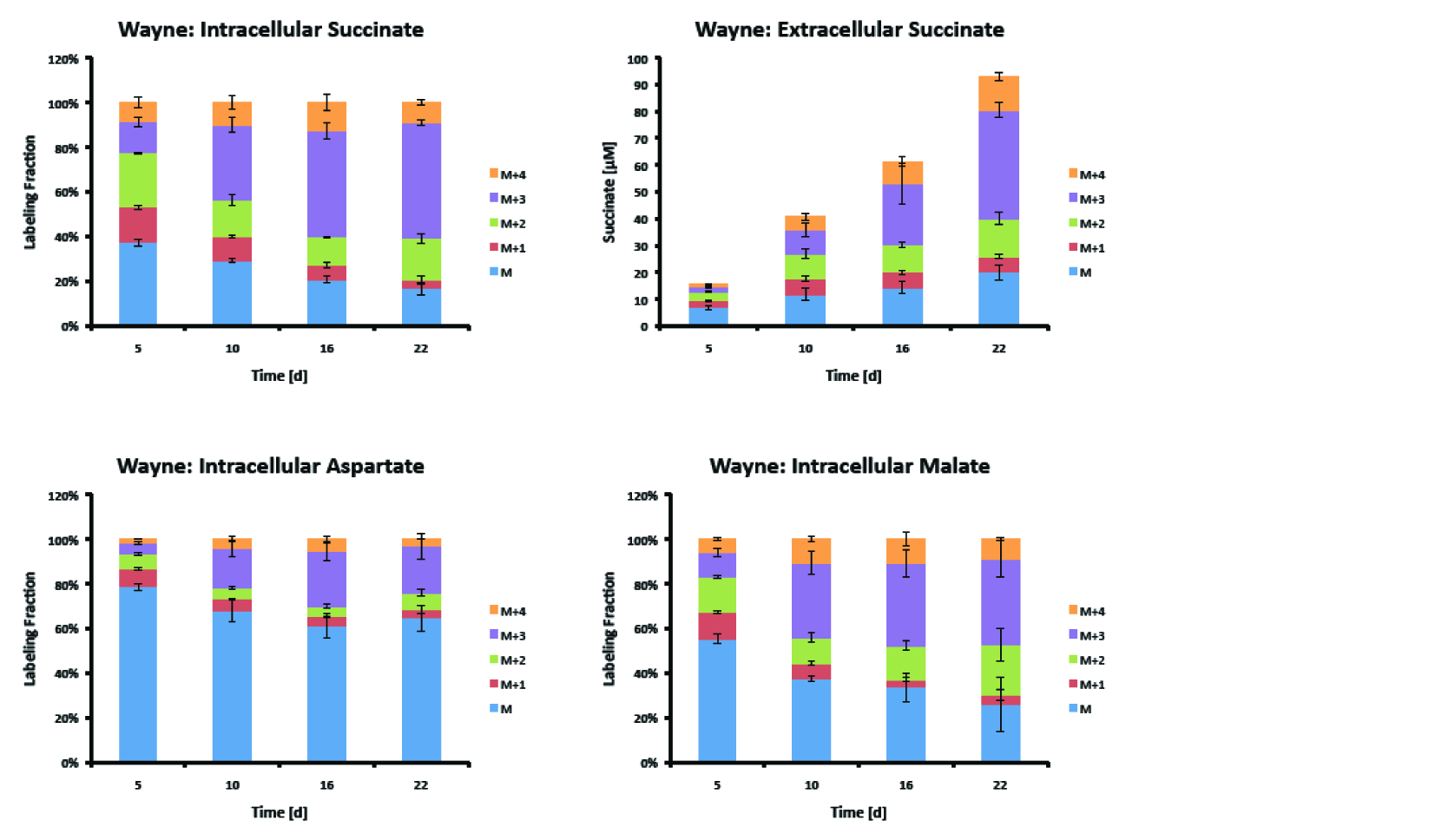

Supplement: Figure S4 — Intracellular and extracellular isotopomer distribution of metabolites of the C4 branch of the TCA cycle of NRP cultures in U-13C Glucose Dubos medium. H37Rv was adapted to hypoxia in the Wayne model using Dubos medium where the glucose had been replaced with U- 13C glucose. Intracellular and extracellular metabolites were analyzed at the indicated time points. Only significant levels of succinate could be detected in the extracellular medium. (TIF) [file ppat.1002287.s004.tif]

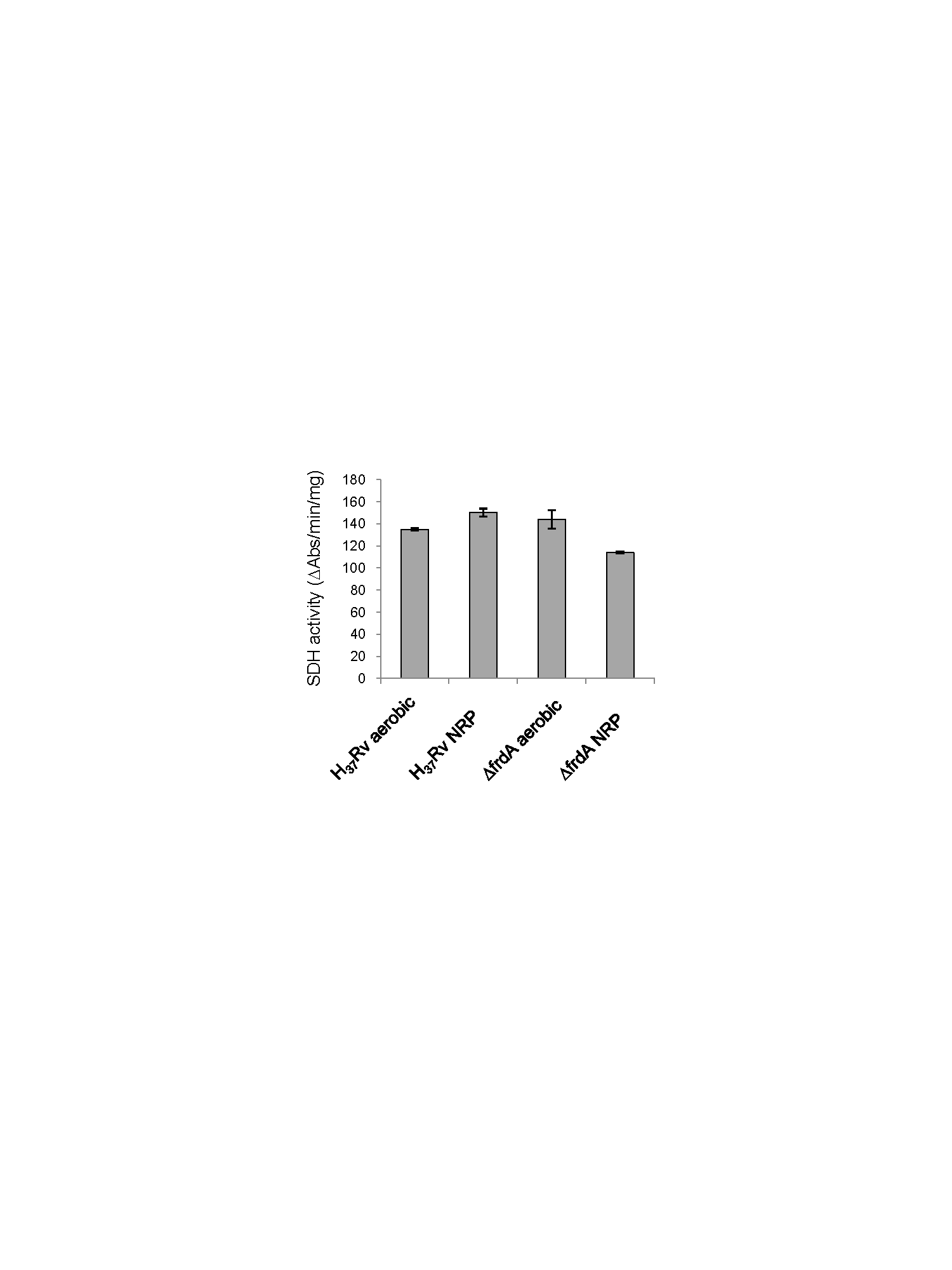

Supplement: Figure S5 — Membrane associated succinate dehydrogenase activities of wild-type or fumarate reductase knockout strains adapted to aerobic or anaerobic conditions. Membrane fractions were prepared from H37Rv and H37Rv ΔfrdA grown aerobically in Dubos medium or adapted to anaerobic conditions in Wayne model tubes followed by measurement of succinate dehydrogenase activities. (TIFF) [file ppat.1002287.s005.tiff]

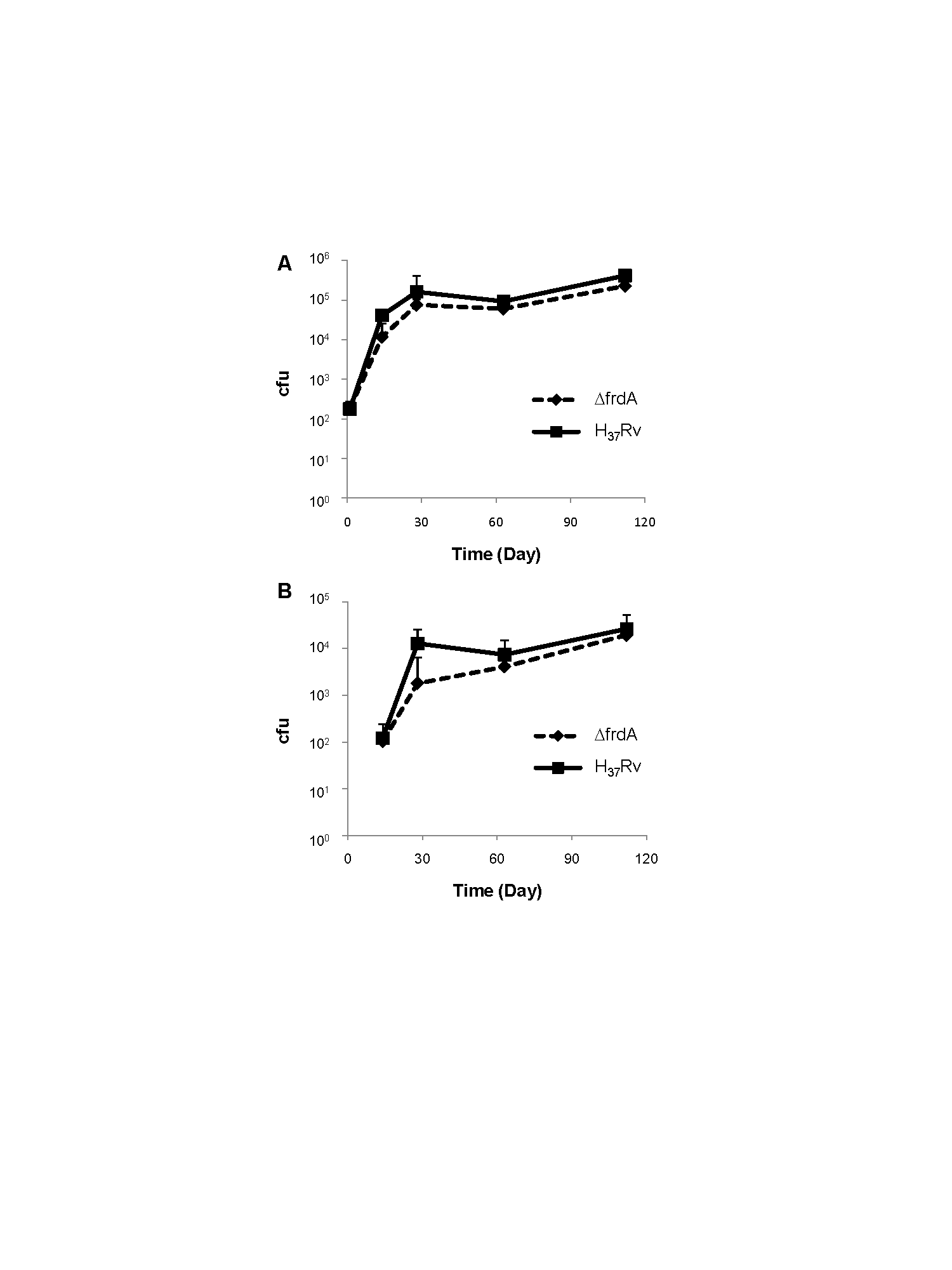

Supplement: Figure S6 — Growth and survival of wild-type or Δ frdA mutant in vivo . Lungs C57Bl/6 mice were infected with 100 CFU of the wild-type and ΔfrdA strains followed by monitoring of bacterial burdens in (A) lungs and (B) spleens in mice over time. Each time point represents the median CFU and standard error of 5 mice per group. (TIFF) [file ppat.1002287.s006.tiff]
